# Transcription of biochemical defenses by the harmful brown tide pelagophyte, *Aureococcus anophagefferens*, in response to the protozoan grazer, *Oxyrrhis marina*

**DOI:** 10.3389/fmicb.2023.1295160

**Published:** 2023-12-13

**Authors:** Walter Dawydiak, Christopher J. Gobler

**Affiliations:** School of Marine and Atmospheric Sciences, Stony Brook University, Southampton, NY, United States

**Keywords:** harmful algal bloom, Brown tide, zooplankton grazing, induced defense, gene express

## Abstract

*Aureococcus anophagefferens* is a small marine pelagophyte that forms recurrent harmful brown tides blooms with adverse ecological and economic impacts. During blooms, *A. anophagefferens* experiences lower zooplankton grazing mortality than other phytoplankton potentially due to the synthesis of anti-predator compounds including extracellular polysaccharides. This study characterized the transcriptomic response of *A. anophagefferens* when exposed to the protozooplankton, *Oxyrrhis marina*, and assessed whether this response involved chemical cues. Transcriptomes were generated from *A. anophagefferens* populations grown at high (1×10^6^ cells mL^−1^) and low (5×10^5^ cells mL^−1^) cell densities incubated directly with *O. marina* or receiving only filtrate from co-cultures of *A. anophagefferens* and *O. marina* to evaluate the role of chemical cues. There were a greater number of genes differentially expressed in response to grazing in the lower concentration of *A. anophagefferens* compared to the high concentration treatment and in response to direct grazing compared to filtrate. KEGG pathway analysis revealed that direct grazer exposure led to a significant increase in transcripts of genes encoding secondary metabolite production (*p* < 0.001). There was broad transcriptional evidence indicating the induction of biosynthetic pathways for polyketides and sterols in response to zooplankton grazers, compounds associated with damage to marine organisms. In addition, exposure to *O. marina* elicited changes in the abundance of transcripts associated with carbohydrate metabolism that could support the formation of an extracellular polysaccharide matrix including genes related to glycoprotein synthesis and carbohydrate transport. Collectively, these findings support the hypothesis that *A. anophagefferens* can induce biochemical pathways that reduce grazing mortality and support blooms.

## Introduction

Harmful algal blooms (HABs) occur when a phytoplankton population proliferates within an ecosystem to a level that causes adverse public health and/or ecological effects (Smayda, 1997a). Some HABs release or contain chemicals that are toxic to other organisms such as shellfish, finfish, and humans (Smayda, 1997b), while others achieve high densities that become destructive to the ecosystem through shading of submerged aquatic vegetation, formation of hypoxic conditions, fish kills, or other harm to marine life (Anderson et al., 2021). Due to these broad detrimental effects, HABs as a group are a human health and environmental concern that can also have a significant economic impact through the closure and decimation of fisheries (Anderson et al., 2021).

Generally, blooms of phytoplankton species occur when their growth rate outpaces their loss rate, allowing for the accumulation of cells (Smayda, 1997a). The growth rate of bloom-forming phytoplankton can be accelerated by changes in temperature, light, nutrient availability, competition, physical mixing, and other factors (Smayda, 1997b). Blooms may also intensify through a reduction in mortality, particularly when grazing by shellfish and zooplankton is inhibited (Smayda, 2008; Anderson et al., 2021).

*Aureococcus anophagefferens* is a small (2–3 microns) marine pelagophyte that causes annually recurring blooms, known as brown tides, in estuaries in the United States, China, and South Africa (Gobler et al., 2011). Brown tides achieve high densities that reduce light penetration, impacting ecologically important seagrass habitat (Cosper et al., 1989; Gobler et al., 2004) and can inhibit feeding in bivalves (Gainey and Shumway, 1991; Bricelj and Lonsdale, 1997). Ample research has been devoted to factors promoting growth rate of *A. anophagefferens*, including the availability of dissolved organic nutrients (Gobler et al., 2004, 2011; Mulholland et al., 2004).

The impacts of loss rates on bloom development in *A. anophagefferens* are not fully clear. Experimental and field observations have shown that *A. anophagefferens* experiences lower grazing mortality than other phytoplankton populations during blooms (Gobler et al., 2002, 2004). Grazers in both field and laboratory communities appear to avoid *A. anophagefferens* when it is present in high concentrations (Caron et al., 2004). During non-bloom conditions, *A. anophagefferens* can experience higher grazing mortality (Deonarine et al., 2006).

While it seems possible that *A. anophagefferens* may initiate a grazing defense to reduce mortality and support the formation of blooms, no mechanism for this has been identified. There are several modes of defense that phytoplankton can use to prevent or limit grazing mortality including the release of toxic or deterrent chemicals, changes to morphology, or life history changes (Lürling, 2021). The production of grazing deterrent compounds in *A. anophagefferens* has long been suspected (Cosper et al., 1989; Buskey and Hyatt, 1995). Its genome contains a relatively high quantity of genes likely related to the production of secondary metabolites, including genes to produce several toxic isoquinalone alkaloids (Gobler et al., 2011). The genome also contains a gene encoding a membrane attack complex, uncommon in most phytoplankton and thought to be involved in defense against protistan grazers (Gobler et al., 2011). *A. anophagefferens* exhibits cytotoxic effects on ribbed mussels (Galimany et al., 2014) and has hemolytic effects on sea bream cells (Huang et al., 2020).

An alternative to the release of secondary metabolites as a grazing defense mechanism is the production of extracellular polysaccharides (EPSs). EPSs create a mucous-like sheath around the cell, imposing a mechanical deterrent to grazing (Sieburth et al., 1988). This mechanism has been shown to reduce grazing rates on a similar brown-tide forming species, *Aureoumbra lagunensis* (Buskey and Hyatt, 1995; Liu and Buskey, 2000) and isolation of the polysaccharide layer from *A. anophagefferens* has shown it can inhibit the ciliary movement of bivalves (Gainey and Shumway, 1991).

Grazer defense mechanisms are likely associated with some degree of cost to the organisms that implement them (Lürling and Van Donk, 2000; Yang et al., 2009; Pančić and Kiørboe, 2018; Park et al., 2023). To limit this cost, some phytoplankton use infochemicals to signal the periodic induction of these defenses only when there is sufficient risk of grazing mortality (Jang et al., 2003), as opposed to employing the defenses constitutively. There are three possible sources for chemicals that could signal the presence of grazers and stimulate a defense response. First, cell lysis or physical damage of prey cells could release alarm substances from conspecific prey cells lysed in the grazing process (Dodson et al., 1994). Conversely, the signal may be a kairomone, or a chemical produced directly by the grazer and not requiring contact or grazing to occur to be detected (Dodson et al., 1994; Lürling and Van Donk, 1997; Senft-Batoh et al., 2015). Lastly, the cue could be produced only when prey cells come in direct physical contact with a feeding grazer (Van Donk et al., 2011). Any of these sources may be used in informing the prey species that there is a risk of grazing mortality and promoting an induction of defense mechanisms.

This study documented the transcriptional response of *A. anophagefferens* to grazing by the protozoan grazer, *Oxyrrhis marina*. *Oxyrrhis marina* is known to co-occur with and graze *A. anophagefferens* (Caron et al., 2004) and has been identified before, during, and after recent blooms in estuaries of Long Island, NY (Deonarine et al., 2006). Grazing by *O. marina* has been shown to stimulate the production of deterrent chemicals in other phytoplankton (Wolfe and Steinke, 1996). *Aureococcus anophagefferens* was co-cultured with *O. marina* in a controlled laboratory experiment and, in addition, filtrate from flasks containing a combination of these organisms was added to flasks containing only *A. anophagefferens* to explore how dissolved constituents produced by the grazing process might elicit a deterrence response in *A. anophagefferens* without direct exposure to zooplankton. The goal of the experiment was to characterize and explore the transcriptional response of *A. anophagefferens* to the direct presence of grazers as well as to any signaling chemicals released in the presence of grazers. We hypothesized that *A. anophagefferens* would induce a defense response when exposed both directly and indirectly to *O. marina* and that the defense response would be more intense with a higher concentration of *A. anophagefferens* and direct exposure to *O. marina*.

## Methods

### Culture maintenance

*Aureococcus anophagefferens* CCMP strain 1850 and *O. marina* CCMP strain 3375 stock cultures were maintained in GSe medium made from 0.2-μm filtered seawater collected from the coastal Atlantic Ocean and with a final salinity of 32 ppt. *A. anophagefferens* CCMP strain 1850 has been shown to have retained its ability to inhibit bivalve grazing after other strains had lost that ability (Harke et al., 2011). Cultures were kept in an incubator at 21°C on a 14:10 light:dark cycle receiving 100 μmol photons m^−2^ s^−1^. *O. marina* was fed 3 × 10^4^ cells mL^−1^ of *Tisochrysis lutea* (Bendif et al., 2013) biweekly. *T. lutea* was maintained at the same light and temperature in f/2 media made with 0.2-μm filtered seawater collected from the coastal Atlantic Ocean and with a final salinity of 32 ppt. The *A. anophagefferens* stock culture was enumerated on a Becton Dickinson Cytoflex flow cytometer. Lugol’s preserved samples from the *O. marina* stock culture were quantified on a Sedgewick rafter counting slide mounted on a light microscope. Initial concentrations for both stock cultures were used to calculate requisite volumes of the stock cultures to achieve the starting concentration targets for each treatment group. Stock cultures were diluted twice weekly to maintain cultures in a state of exponential growth.

### Experimental design and processing

**Figure 1:**
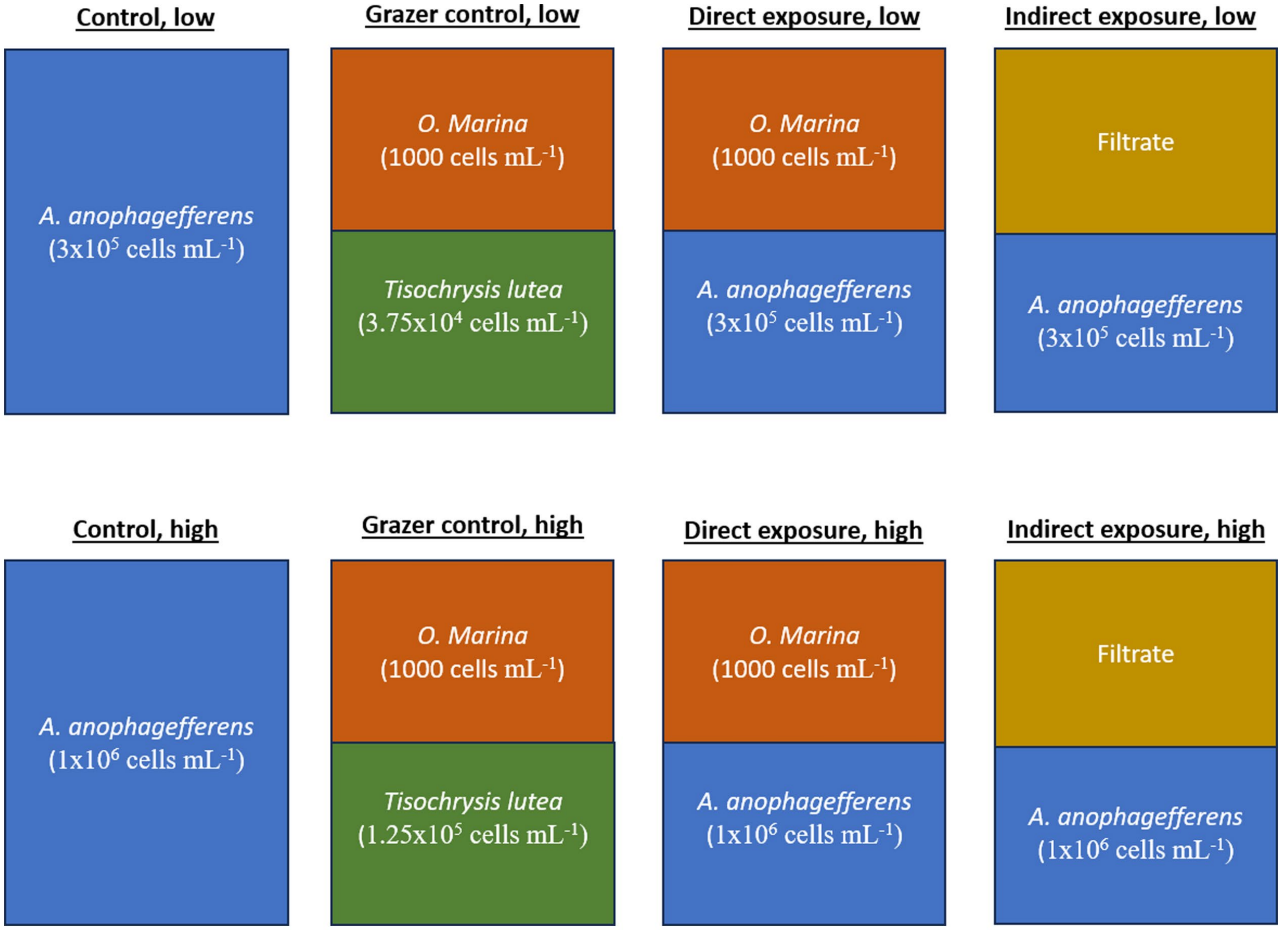
Diagram of treatments used in the experiment.

The experiment presented here consisted of four experimental treatments and two control treatments (Figure 1). The experimental treatments included a “direct grazing” treatment with *A. anophagefferens* and *O. marina* in the same flask as well as an “indirect grazing exposure” treatment where flasks containing only *A. anophagefferens* received filtrate from additional, direct grazing flasks. The control treatment contained only *A. anophagefferens*. These three groups were incubated at low (5 × 10^5^ cells mL^−1^) and high concentrations (1×10^6^ cells mL^−1^) of *A. anophagefferens* to simulate early and peak bloom conditions, respectively (Caron et al., 2004). The concentration of the *A. anophagefferens* stock cultures were approximately 1×10^6^ cells mL^−1^ for the low concentration treatments and 2 × 10^6^ cells mL^−1^ for the high concentration treatments. An additional “grazer control” group was created in a similar fashion to the direct grazing treatments, with *O. marina* mixed with an “ideal prey,” *T. lutea,* to generate comparative grazing rates by the same grazers (Figure 1). *T. lutea* was added to the grazer control treatments at an equivalent biovolume to *A. anophagefferens* (Harke et al., 2011). The starting concentrations of *T. lutea* were 6.25×10^4^ cells mL^−1^ for the grazer control, low group and 1.25×10^5^ cells mL^−1^ in the grazer control, high group. The concentration of the *T. lutea* stock culture was approximately 1.5×10^6^ cells mL^−1^. All groups were produced in triplicate 500 mL Erlenmeyer flasks.

*Oxyrrhis marina* was of 1,000 cells mL^−1^. The *O. marina* stock culture was starved prior to the experiment and concentrated on a 10-μm sieve to prevent the transfer of *T. lutea* cells into experimental flasks. The *O. marina* stock culture concentration was approximately 6.8 × 10^3^ cells mL^−1^. Calculated volumes of the organisms were added to flasks and topped off with GSe to bring the total volume to 240 mL. Flasks were incubated at 21°C on a platform rotating at 50 rpm.

To prevent disturbing the direct grazing flasks in collecting filtrate for the indirect grazing treatment, a set of “donor” flasks was created in duplicate exactly mimicking the direct grazing flasks, using the same concentrations of the two study organisms and incubated under the same conditions throughout the experiment. This served to mimic the grazing conditions occurring in the direct grazing flasks while providing a source for filtrate so that the direct grazing flasks were not manipulated. At 8 h, 12 h, and 22 h, 20 mL were removed from each donor flask and filtered through a sterile 0.22-μm bottle-top filter and added to the indirect grazing flasks. All other flasks concurrently received 20 mL of GSe to maintain volumetric consistency between treatments. Quantification of *A. anophagefferens* and *O. marina* within donor flasks along with the control and experimental treatments confirmed comparability to the direct grazing treatment flasks. No RNA samples were collected from the donor flasks.

After 24 h of incubation, Lugol’s samples were collected for *O. marina* counts. Separate samples were preserved in glutaraldehyde for flow cytometric counts *of A. anophagefferens*. Growth rates were calculated as Growth rate (d^−1^) = ln(((final concentration*dilution factor)/initial concentration)/(days elapsed)). A dilution factor of 1.25 was used to correct the final *A. anophagefferens* concentrations for the 60 mL of total GSe or filtrate added to each flask throughout the experiment. To capture cells for RNA isolation, 20 mL of water from each flask were filtered through Millipore Sigma Isopore 0.2-μm polycarbonate membrane filters. Direct exposure flasks containing both *O. marina* and *A. anophagefferens* were passed through a 10-μm sieve separating *A. anophagefferens* from *O. marina* before collection on filters. Microscopic examination affirmed this effectively separated the two eukaryotes. After filtration, filters were placed into 2.0-mL microcentrifuge tubes that were immediately flash frozen in liquid nitrogen. Care was taken to ensure the study organisms in each individual flask went from flask to flash freezing in less than 3 min. Frozen filters were stored at −80°C until RNA extraction.

Extraction of RNA was carried out using a Zymo Research Quick-RNA Fungal/Bacterial Miniprep Kit following the manufacturer’s instructions. Contaminating genomic DNA was digested using Qiagen RNase-free DNase following the manufacturer’s instructions. Purified RNA was analyzed for quality using a ThermoScientific NanoDrop One Spectrophotometer and an Agilent 2100 BioAnalyzer. The RNA samples were then sent to the J.P. Sulzberger Columbia Genome Center for sequencing on an Illumina HiSeq 2500 with a target sequencing depth of 20 million 50-base pair (bp) paired-end reads per sample.

### Bioinformatic analyses

Raw reads were checked for quality using FastQC (Version 0.11.6, Andrews, 2010) and then underwent light quality trimming using Trimmomatic (Version 0.36, Bolger et al., 2014). Trimmomatic settings included a minimum read length of 36, a sliding window of 5 through 20, leading and trailing quality of 3, and trimming of adapter sequences. The trimmed reads were aligned to a strain 1850 reference genome (Gann et al., 2021) using STAR (Version 2.5.3a, Dobin et al., 2013). These alignments were used to create a summary count table for each replicate in RSEM (Version 1.3.0, Li and Dewey, 2011). Finally, counts for each annotated gene were compiled for all replicates for analysis in DESeq2 (Version 1.32.0, Love et al., 2014). Transcript abundance for all genes annotated in the reference genome was compared between control and experimental treatments to identify the transcriptional response of *A. anophagefferens* to direct and indirect grazing exposure.

DESeq2 normalizes samples by library size, estimates dispersion for each gene, and shrinks these dispersion estimates. A negative binomial generalized linear model is then applied to each gene and a Wald test is performed to test whether the log_2_ fold change between the experimental and control group is significantly different than zero. DESeq2 provides log_2_ fold change and Benjamini-Hochberg adjusted *p* values for each gene which were used in subsequent visualizations and analyses.

Expression results from DESeq2 were used to perform pathway enrichment analysis using the GAGE package (Luo et al., 2009) and the Kyoto Encyclopedia of Genes and Genomes (KEGG) database. Pathway enrichment analysis groups genes into linked functional pathways and determines whether these biochemical pathways underwent up- or downregulation as a result of the experimental stimulus. This provides a more meaningful system-level view of expression results that may be more informative than simply evaluating expression of many individual genes on their own.

Gene set enrichment analysis (GSEA) was carried out with the expression data and the Gene Ontology (GO) database using the topGO package (Alexa et al., 2006). Terms within the GO database are organized into three subontologies which are Biological Process (BP), Molecular Function (MF), and Cellular Component (CC). The MF subontology includes the most rudimentary processes at the molecular level and are not annotated with specificity to any larger individual process or organelle. BP terms relate to broader, more complex activities involving multiple steps such as the response to high light intensity. The CC subontology describes where in the cell activity is occurring, such as within the chloroplast or cytosol.

## Results

**Figure 2:**
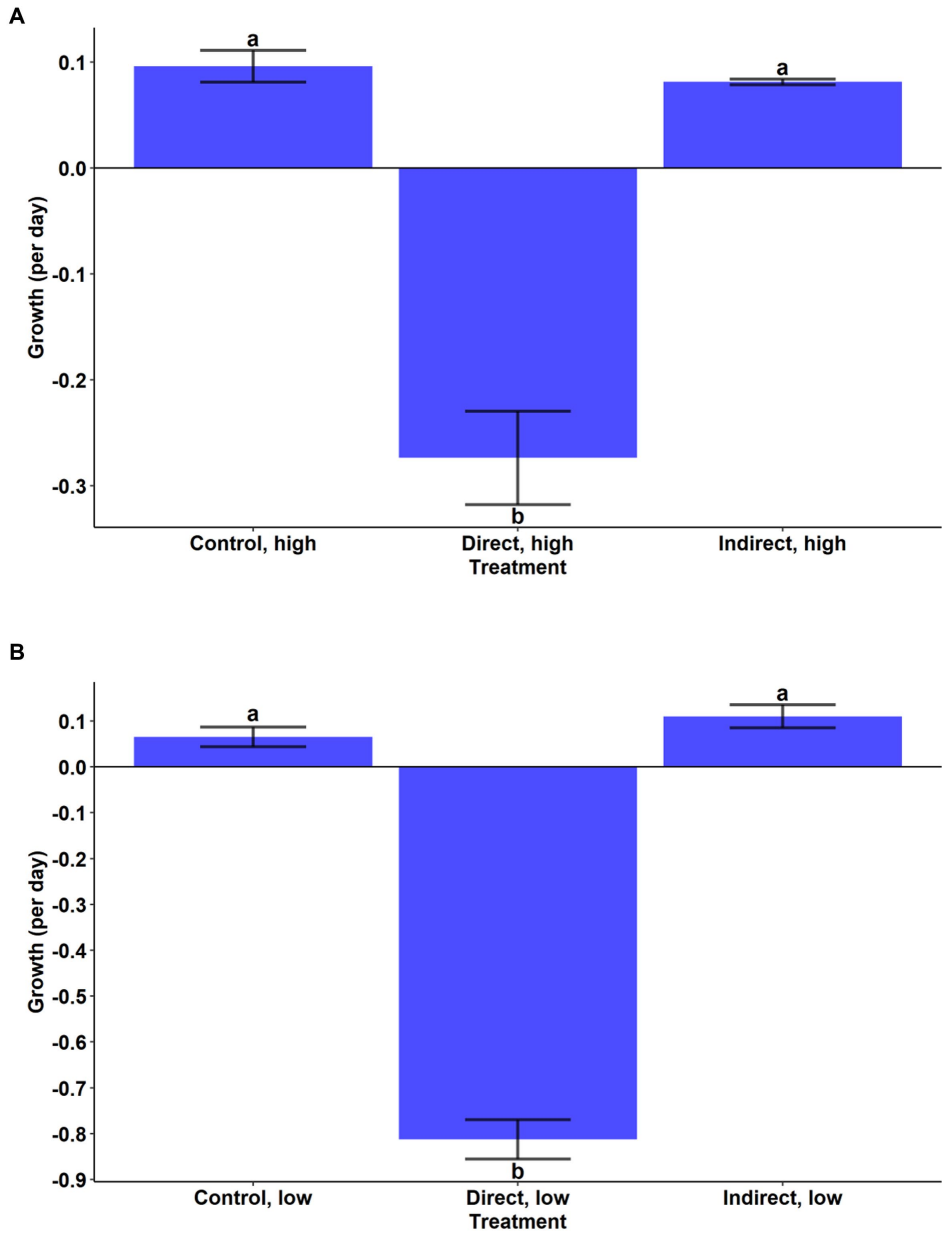
Cellular net growth rate (per day) of *A. anophagefferens* in the **(A)** high cell density treatments and **(B)** low cell density treatments. Bars are means while error bars show standard deviation of the mean of the three replicates in each treatment. Letters indicate significantly different mean growth rates (Tukey HSD).

**Figure 3:**
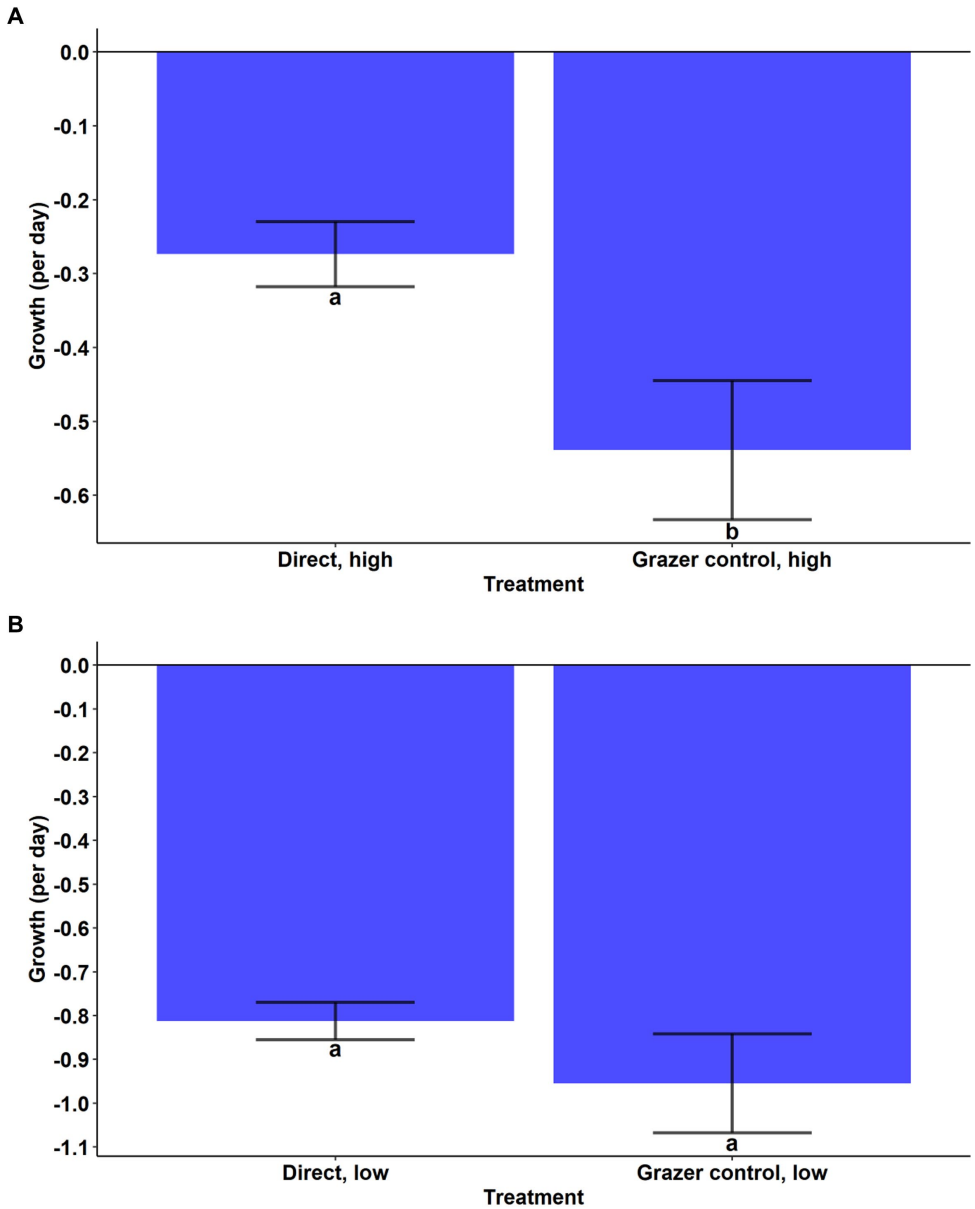
**(A)** Cellular net growth rates (per day) of *Aureococcus anophagefferens* (Direct, high) and *Tisochrysis lutea* (Grazer control, high). Significance by Welch’s *t*-test. **(B)** Cellular net growth rates (per day) of *Aureococcus anophagefferens* (Direct, low) and *Tisochrysis lutea* (Grazer control, low). Significance by Welch’s *t*-test.

During the experiment, *A. anophagefferens* experienced a reduction in cell densities in the high and low concentration direct grazing treatments while the control and indirect groups both displayed positive growth (Figures 2A,B). Growth rates in the high and low direct grazing treatments were significantly lower than in the respective control and indirect treatments (Figures 2A,B, *p* < 0.001, Tukey’s test). The mortality rate of *A. anophagefferens* in the direct, high group was significantly lower than that of *T. lutea* in the grazer control, high group (Figure 3A, Welch’s *t*-test, *p* = 0.0031) and was marginally lower in the direct, low group (Figure 3B, Welch’s *t*-test, *p* = 0.0594).

**Figure 4:**
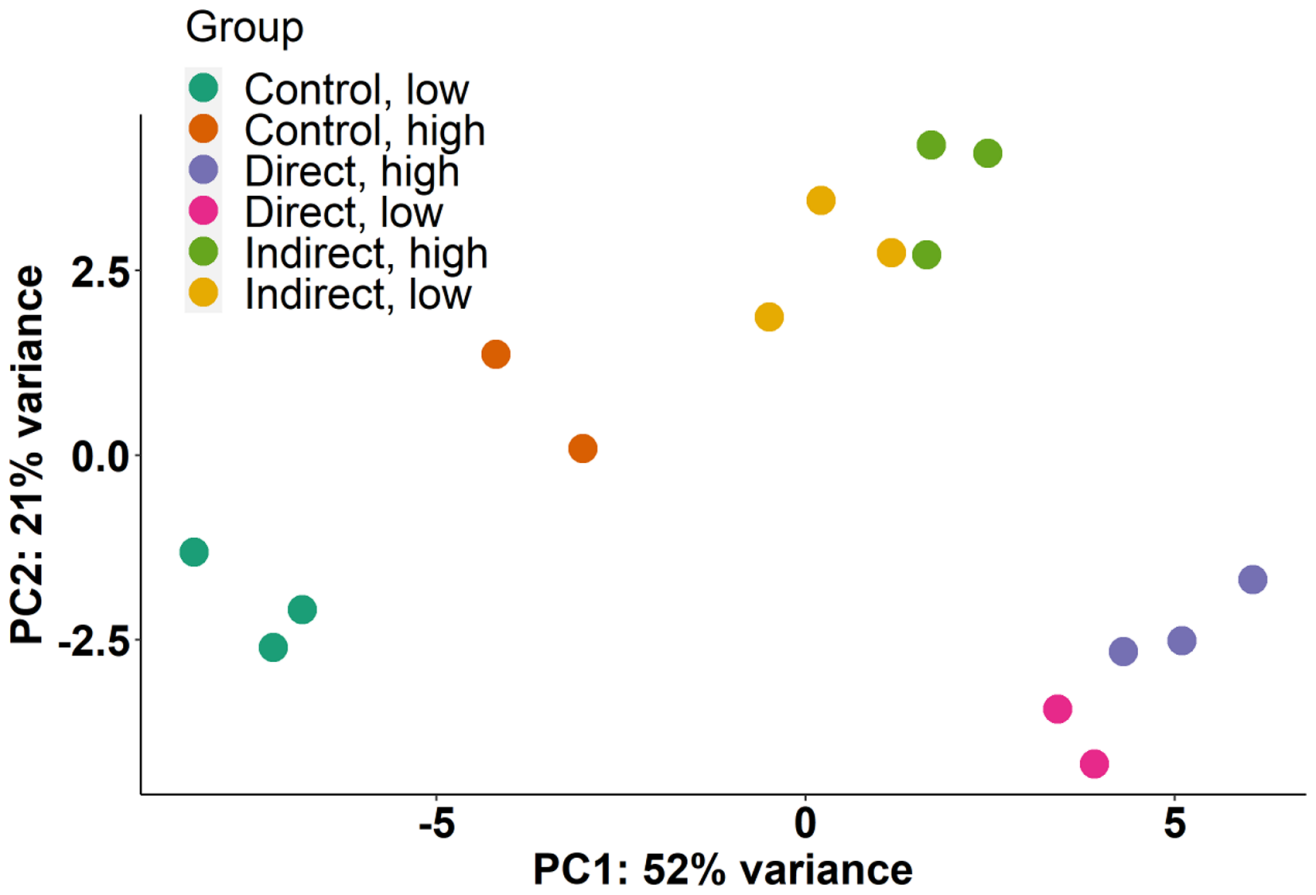
Principal-component analysis (PCA) of normalized read counts for each replicate. Percentages on each axis represent the percent of variance explained.

Transcriptome sequencing produced libraries ranging in size from ~10 million to ~34 million 50-bp paired end reads for each replicate. Following quality trimming, alignment rate to the CCMP strain 1850 reference genome ranged from 28.62 to 83.21% (Supplementary Table 1). Principal-component analysis (Figure 4) was used to explore the relatedness of transcriptomes from each treatment. The direct, high and direct, low treatments clustered together, as did the indirect, high and the indirect, low treatments. Most variability in expression patterns (PC1 = 52% variance) was accounted for by a separation of the direct exposure treatments and the control treatments, with the indirect exposure treatments falling roughly in between these two along PC1 (Figure 4). Most of the variance explained in PC2 (21% variance) appeared to be driven by the difference between the expression in the indirect treatments compared to the control and direct exposure treatments.

**Figure 5:**
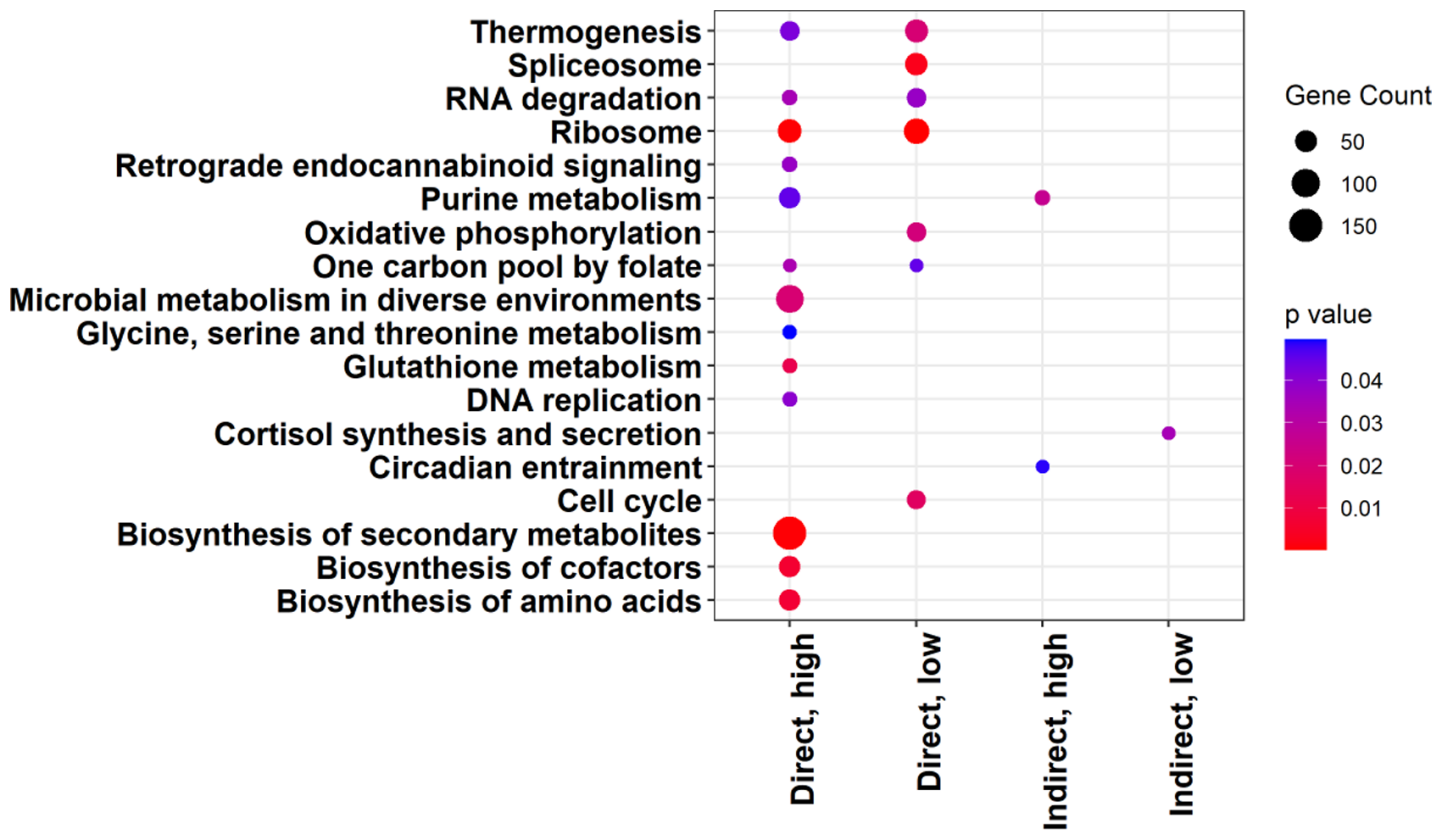
Significantly upregulated KEGG pathways with a *p* value of less than 0.05 for each treatment. Gene count represents the number of significant genes in each pathway for each treatment.

**Figure 6:**
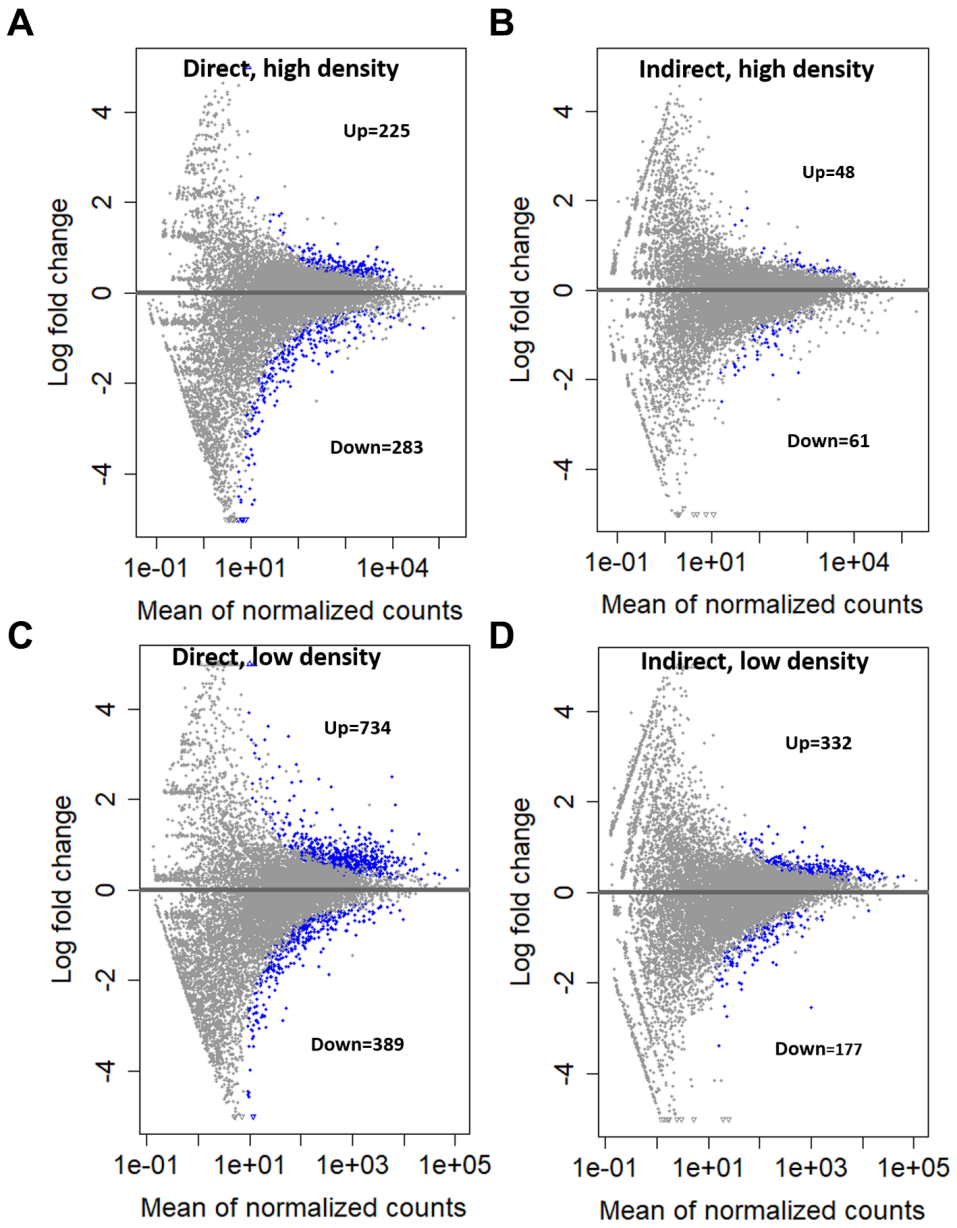
Mean expression vs. log fold change (MA plot) compared to the control treatment for **(A)**. Direct grazing, high cell density, **(B)** Indirect grazing, low cell density, **(C)** Direct grazing, low cell density, **(D)** Indirect grazing, low cell density. Significant genes (FDR = 0.05) shown in blue, non-significant in gray.

KEGG pathway enrichment analysis indicated the largest number of significantly upregulated pathways occurring in the direct, high group (Figure 5). The direct exposure treatments both yielded more significantly upregulated pathways than their indirect exposure counterparts (Figure 5; direct, high = 13; indirect, high = 2; direct, low = 7; indirect, low = 1). The direct exposure treatments also had more significantly enriched gene sets in the GO analysis (Supplementary Figures 1–3). Similarly, there were more genes with an increase in transcript abundance in the direct exposure treatments compared to the indirect treatments (Figure 6A, direct, high: up = 225, down = 283; Figure 6B, indirect, high: up = 48, down = 61; Figure 6C, direct, low: up = 734, down = 389; Figure 6D, indirect, low: up = 332, down = 177; FDR = 0.05). In contrast to the KEGG results, the overall number of differentially expressed genes was lower for each of the high concentration groups (Figure 6A, direct = 508; Figure 6B, indirect = 109) than for the associated low concentration groups (Figure 6C, direct = 1,123; Figure 6D, indirect = 509).

### Polyketides and fatty acids

Gene ontology (GO) analyses revealed that there were several significantly enriched pathways related to polyketide and fatty acid synthesis terms in all treatments (Supplementary Table 2). Significant terms (*p* < 0.05) within this subset in the direct, high treatment included cellular response to fatty acid (biological process; BP), S-adenosylmethionine cycle (BP), S-adenosylmethionine-dependent methyltransferase activity (molecular function; MF), and [acyl-carrier-protein] S-malonyltransferase activity (MF; Supplementary Table 2). In the indirect, high group, terms for positive regulation of fatty acid oxidation (BP), fatty acid binding (MF), and diacylglycerol O-acyltransferase activity (MF) were enriched (Supplementary Table 2). In the direct, low treatment, enriched terms related to fatty acid metabolism included “fatty acid elongation, saturated fatty acid” (BP), “fatty acid elongation, monounsaturated fatty acid” (BP), “fatty acid elongation, polyunsaturated fatty acid” (BP), and fatty acid elongase activity (MF; Supplementary Table 2). Also, acetyl-CoA C-acyltransferase activity (MF) was enriched in the indirect, low treatment (Supplementary Table 2).

At the gene level, there were multiple genes involved in polyketide and fatty acid synthesis that displayed significant increases in transcript abundance in one or more treatments (Supplementary Table 3). For example, fatty acid elongation, fatty acid desaturase, [acyl-carrier-protein] S-malonyltransferase activity, acetyl-CoA carboxylase, long-chain-3-hydroxyacyl-CoA dehydrogenase activity, very-long-chain 3-ketoacyl-CoA synthase activity, and fatty acid biosynthetic process genes all had significantly higher transcript abundance (Supplementary Table 3; direct, high = 4; indirect, high = 2; direct, low = 13; indirect, low = 12; *p* < 0.05). Other genes putatively associated with polyketide synthesis with significant or marginally significant changes in transcript abundance included polyketide synthase modules and related proteins (Gene ID: JL721_1454, direct, low, *p* = 0.0970, log_2_ fold change = 0.33), antibiotic biosynthesis monooxygenase (Gene ID: JL721_3539 and Gene ID: JL721_2332, direct, low, multiple genes, *p* < 0.01, log_2_ fold change from 0.61 to 0.79; indirect, low, *p* = 0.0811, log_2_ fold change = 0.34), macrocin-O-methyltransferase (TylF) (Gene ID: JL721_3539, direct, low, *p* = 0.0801, log_2_ fold change = −1.47; indirect, low, *p* = 0.0341, log_2_ fold change = −1.71), and phosphopantetheine binding (Supplementary Table 3; direct, low, Gene ID: JL721_11201, *p* = 0.0252, log_2_ fold change = −1.77; direct, low, Gene ID: JL721_4843, *p* = NA, log_2_ fold change = 4.94; indirect, low, Gene ID: JL721_3913, *p* = 0.0034, log_2_ fold change = 0.45). An S-adenosyl-L-methionine transmembrane transport gene had significantly lower transcript abundance in all treatments (Supplementary Table 3; all treatments, Gene ID: JL721_837, log_2_ fold change from −0.76 to −1.70; Gene ID: JL721_2862, direct, high, log_2_ fold change = −1.51; *p* < 0.05). Another gene that catalyzes the formation of S-adenosylmethionine from methionine and ATP displayed higher transcript abundance in all treatments except for indirect, high (Gene ID: JL721_6224, Supplementary Table 3; *p* < 0.01, log_2_ fold change from 0.40 to 0.84). Also, a gene encoding a protein involved in the biosynthesis of the polyketide toxin fumonisin was upregulated with log_2_ fold changes ranging from 0.38 to 0.52 and adjusted *p*-values below 0.1 in all treatments except in the indirect, high treatment (Gene ID: JL721_10524; Supplementary Table 3).

### Sterols

Differential expression related to sterols, steroids, and hormone chemicals appeared in all analyses. KEGG pathway enrichment analysis showed that the pathway for cortisol synthesis and secretion was upregulated in the indirect, low (*p* = 0.0345) treatment and was marginally upregulated in the indirect, high (*p* = 0.0542) treatment (Supplementary Table 4). Within the GO analysis, several terms related to sterols were significantly enriched (Supplementary Table 2, *p* < 0.05) including response to growth hormone (direct, high and direct, low, BP), response to peptide hormone (direct, high, BP) hormone-mediated signaling pathway (direct, high, BP), (17Z)-protosta-17(20),24-dien-3beta-ol biosynthetic process (indirect, high, BP), lanosterol synthase activity (indirect, high, MF), sterol 14-demethylase activity (indirect, high, MF), sterol regulatory element binding protein cleavage (direct, low, BP), and positive regulation of glucocorticoid receptor signaling pathway (indirect, low, BP).

Each treatment had multiple genes related to sterol activity with significant increases in transcript abundance relative to the control including sterol 14-demethylase activity, sterol 24-C-methyltransferase activity, sterol 14-demethylase activity, and C-4 methylsterol oxidase activity (Supplementary Table 5; direct, high = 3, indirect, high = 3, direct, low = 4, indirect, low = 2, *p* < 0.05). Among this subset, sterol 14-demethylase activity showed the highest fold change, especially in the direct, low (log_2_ fold change = 0.89) and indirect, low (log_2_ fold change = 1.10) treatments (Gene ID: JL721_4065; Supplementary Table 5, *p* < 0.05). Other genes involved in sterol biosynthetic pathways with a significant increase in transcript abundance included dimethylallyltranstransferase activity (Gene ID: JL721_4942, direct, low, *p* = 0.0081; indirect, low, *p* = 0.0500) and isopentenyl-diphosphate delta-isomerase activity (Gene ID: JL721_5756, direct, low, *p* = 0.0293; Supplementary Table 5). Genes for isoprenoid synthesis and 2-C-methyl-D-erythritol 2,4-cyclodiphosphate synthase activity displayed marginally higher transcript abundance in the indirect, low treatment (*p* < 0.1; Supplementary Table 5). Activity related to quinone was observed in genes encoding sulfide:quinone oxidoreductase activity (Gene ID: JL721_11208, direct, high, *p* = 0.0116), NADH dehydrogenase (quinone) activity (Gene ID: JL721_10006, direct, low, *p* = 0.0155), and quinone binding (Gene ID: JL721_5322, direct, high, *p* = 0.0542; Supplementary Table 5). There were multiple genes associated with hormones and steroids that yielded significantly higher transcript abundance compared to the control including 3-beta-hydroxy-delta5-steroid dehydrogenase activity (Gene ID: JL721_12311, direct, low, log_2_ fold change = 0.82, *p* < 0.001; indirect, low, log_2_ fold change = 0.39, *p* = 0.0273), 13-prostaglandin reductase activity (Gene ID: JL721_4141, direct, high, *p* = 0.0937), prostaglandin-F synthase activity (Gene ID: JL721_1110, direct, low, *p* = 0.0379; indirect, low, *p* = 0.0525), and negative regulation of brassinosteroid mediated signaling pathway (Gene ID: JL721_10017, indirect, low, *p* = 0.0650; Supplementary Table 5).

### Polysaccharides

Direct and indirect exposure to *O. marina* significantly altered carbohydrate metabolism in *A. anophagefferens* (Supplementary Table 2; GO analysis; *p* < 0.05). For example, GO terms for GDP-mannose metabolic process (direct, high, BP), sucrose biosynthetic process (direct, high, BP), starch biosynthetic process (direct, high, BP), gluconeogenesis (direct, high, BP), GDP-mannose 3,5-epimerase activity (direct, low, MF), and oligosaccharyltransferase complex (indirect, low, CC) were all enriched (Supplementary Table 2). In all but the indirect, high treatment, GO analysis highlighted significantly increased (*p* < 0.05) activity occurring within the extracellular matrix and cell wall, potentially regions of polysaccharide secretion (Supplementary Table 2).

Multiple genes related to carbohydrate metabolism also displayed differential abundance including pectate lyase activity (Gene ID: JL721_621, direct, high, *p* = 0.0219) and (1- > 6)-beta-D-glucan biosynthetic process (Gene ID: JL721_1276, Supplementary Table 6; direct, high, *p* = 0.0429). There were many genes with increased transcript abundance relative to the control that were related to metabolism of monosaccharides including mannose, xylose, glucose, fructose, pentose, fucose, and galactose (Supplementary Table 6; direct, high = 5; indirect, high = 1; direct, low = 22; indirect, low = 11; *p* < 0.05). Carbohydrate export and transport genes also displayed increased transcript abundance across the treatments (Supplementary Table 6; direct, high = 2; indirect, high = 2; direct, low = 5; indirect, low = 6; *p* < 0.05). Several genes related to polysaccharides displayed particularly high transcript abundance including nucleotide-sugar transmembrane transporter activity (Gene ID: JL721_7942, direct, high, log_2_ fold change = 1.00, *p* < 0.01), mannosyl-oligosaccharide 1,2-alpha-mannosidase activity (Gene ID: JL721_10360, direct, low, log_2_ fold change = 2.46, *p* < 0.01), and pyrimidine nucleotide-sugar transmembrane transporter activity (Gene ID: JL721_4650, Supplementary Table 6; direct, low, log_2_ fold change = 0.96, *p* < 0.05).

### Potential toxins and secondary metabolites

**Figure 7:**
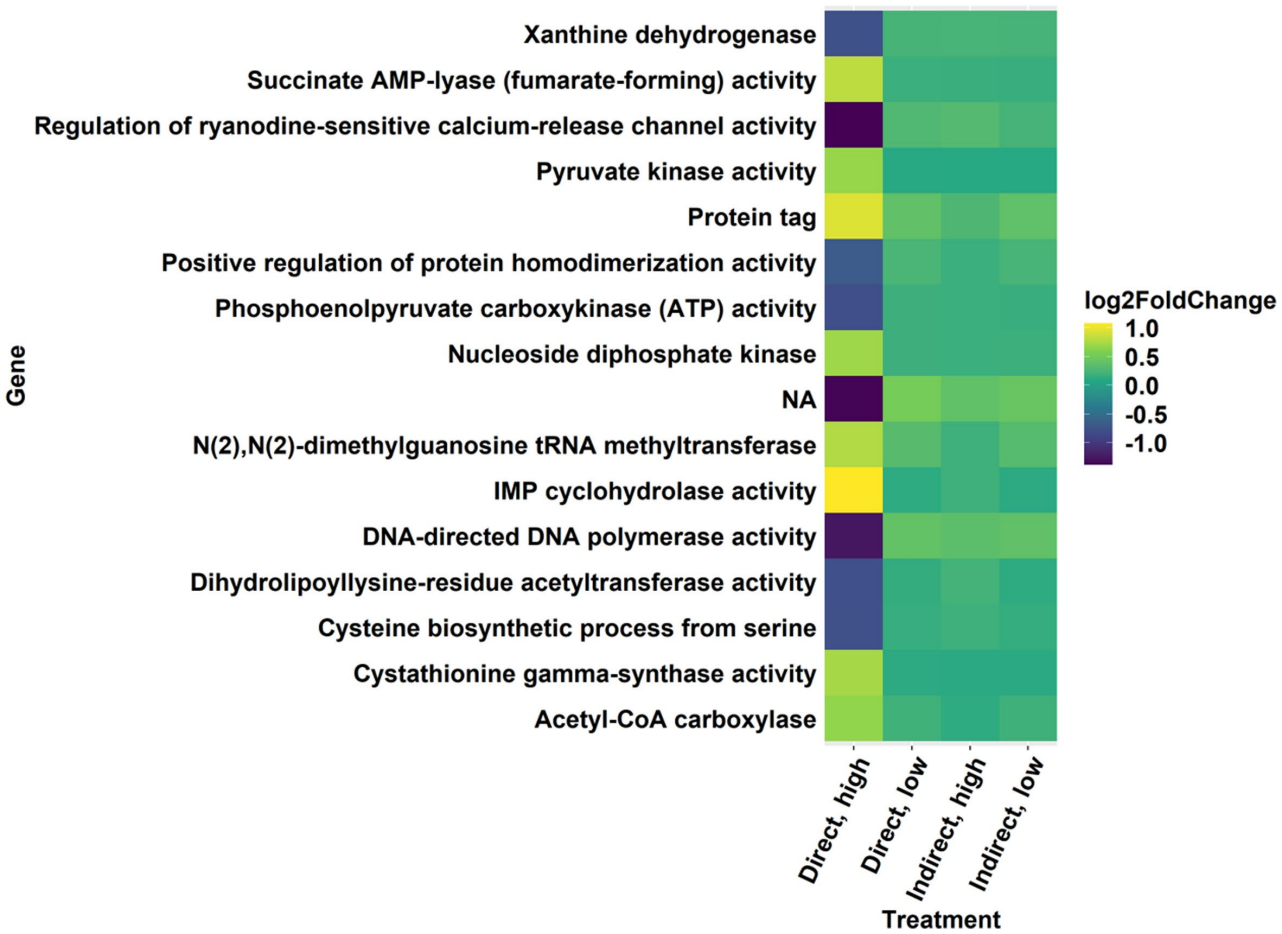
Significantly differentially expressed genes with a log-fold change threshold of ±0.6 in the direct, high treatment from the secondary metabolite KEGG pathway.

In all treatments, there was differential expression of genes in KEGG pathways and GO terms with general annotations referring to toxins, drugs, or antibiotics. The KEGG pathway for biosynthesis of secondary metabolites was upregulated with a high level of significance in the direct, high treatment (Figures 5, 7, *p* < 0.001; Supplementary Figure 4) and was also upregulated with marginal significance in the direct, low treatment (Supplementary Table 4, *p* = 0.0626). Another upregulated pathway potentially related to the synthesis of secondary metabolites was “drug metabolism – other enzymes” (Supplementary Table 4; direct, high, *p* = 0.0708). There were several significantly enriched GO gene sets that referenced toxin and drug activity, especially in the direct, low treatment (Supplementary Table 2; direct, high = 2; indirect, high = 1; direct, low = 6; indirect, low = 3; *p* < 0.05). These terms included antibiotic metabolic process, toxin transport, cellular response to toxic substance, regulation of response to drug, and drug binding (Supplementary Table 2). In the direct, high treatment, a term for induction by symbiont of host defense response was significantly enriched (Supplementary Table 2, *p* = 0.038).

In the differential gene expression analysis, there were multiple general toxin or drug transport genes with significant or marginally significant changes in transcript abundance (Supplementary Table 7). A gene coding for a putative small multi-drug export protein displayed increased transcript abundance in the direct, high treatment (Gene ID: JL721_11702, Supplementary Table 7; log_2_ fold change = 0.83, *p* = 0.1006). In the direct, low treatment, a toxin transport gene had a lower number of transcripts relative to the control (Gene ID: JL721_7974, Supplementary Table 7; *p* = 0.0120). Other toxin-related genes with a reduction in transcript abundance included cytolysis (Supplementary Table 7; Gene ID: JL721_2978; direct, low, *p* = 0.0050; indirect, low, *p* = 0.0080, and Gene ID: JL721_2979; direct, low, *p* = 0.0924; indirect, low, *p* = 0.0641).

### Other pathways

In addition to differential expression related to polyketides, sterols, polysaccharides, toxins, and secondary metabolites, several other categories of pathways were impacted by exposure to grazers. Significantly upregulated KEGG pathways included those related to metabolic activities (microbial metabolism in diverse environments and biosynthesis of cofactors in direct, high; oxidative phosphorylation in direct, low), protein synthesis (ribosome, direct, low, and direct, high; biosynthesis of amino acids, direct, high), and nucleic acid processes (Figure 5; spliceosome, direct, low; RNA degradation, direct low and direct, high; DNA replication, direct, high). There was only one significantly downregulated KEGG pathway across all the treatments (Supplementary Table 4; methane metabolism, indirect, high). The KEGG pathway for retrograde endocannabinoid signaling was significantly upregulated in the direct, high treatment (Figure 5).

## Discussion

In this study, direct and indirect exposure of *A. anophagefferens* to *O. marina* stimulated a broad transcriptional response that may have supported inducible chemical defenses. Although *A. anophagefferens* experienced significant mortality in direct exposure to *O. marina*, it experienced lower mortality compared to *T. lutea*, indicating it experienced reduced grazing mortality (Caron et al., 2004; Deonarine et al., 2006). KEGG pathway enrichment analysis showed that the pathway for biosynthesis of secondary metabolites was upregulated with very high significance for the direct, high treatment. GO analysis revealed significant enrichments of gene sets related to antibiotic and drug processes across all treatments. In addition, all treatments showed upregulation of genes potentially related to the synthesis of secondary metabolites, including polyketides and sterols, compounds known to cause harm to marine organism (Agrawal et al., 2017; Fagundes and Wagner, 2021). While increased transcript abundance was typically not universal for all genes within a given pathway, even partial increased transcription within a pathway can evidence increased protein synthesis (Dyhrman et al., 2012).

### High vs. low cell density treatments

**Figure 8:**
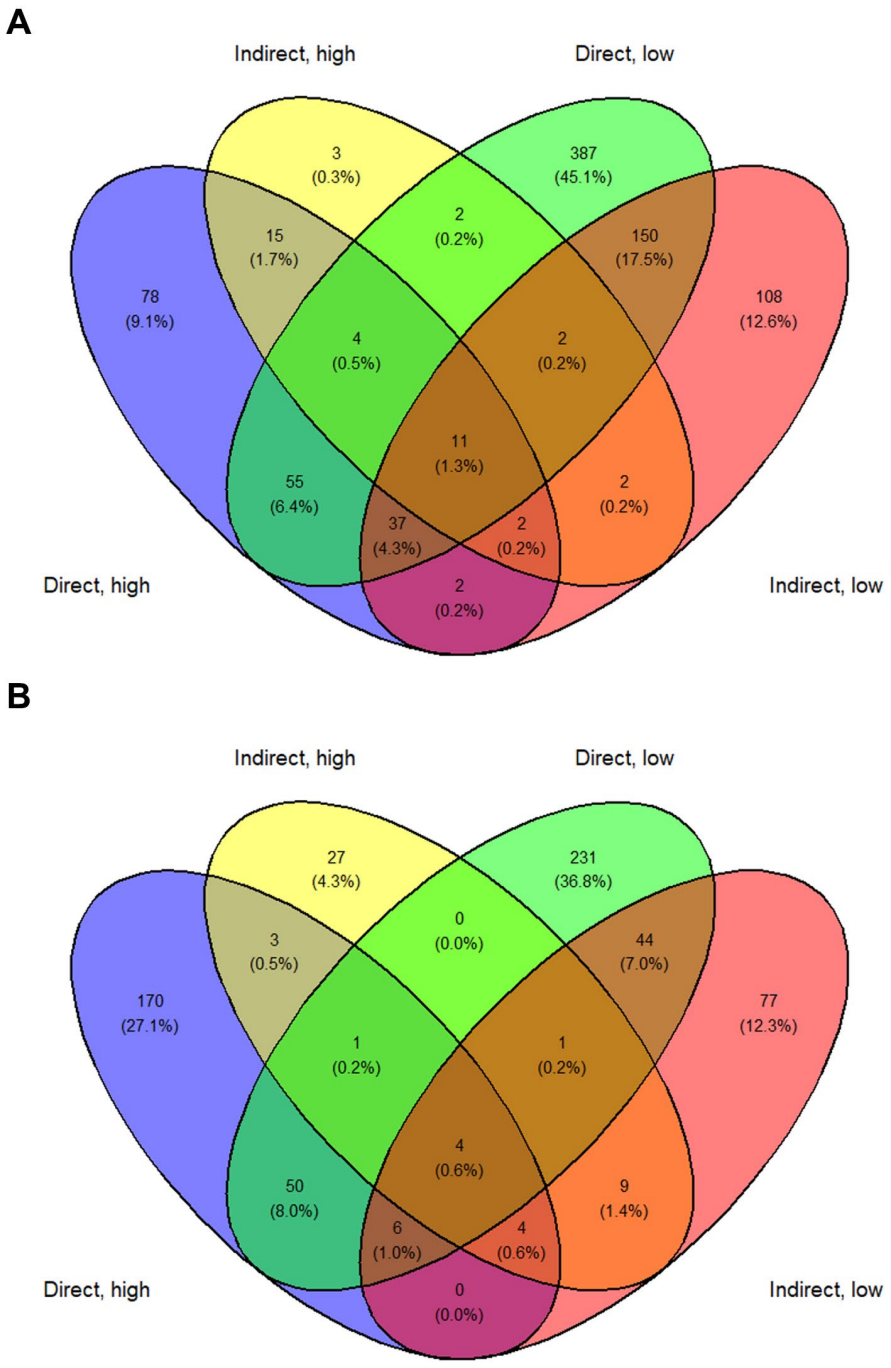
Venn diagram showing **(A)**. Genes with significantly increased transcript abundance and **(B)**. Genes with significantly decreased transcript abundance for each treatment and across treatments.

The direct, low group experienced more grazing mortality as a proportion of population than the direct, high group. This is consistent with past observations wherein grazing mortality decreases with increasing concentration of *A. anophagefferens* and suggests a density dependence in grazing inhibition by *A. anophagefferens* (Caron et al., 2004; Deonarine et al., 2006). Principal component analysis (PCA) revealed that both direct exposure groups clustered together, as did both indirect exposure groups indicating that the difference in starting concentration of *A. anophagefferens* did not result in large differences in gene expression. By other metrics, the response of *A. anophagefferens* exposed to zooplankton grazers was similar, but more intense, in the low concentration treatments. In these treatments, there was a greater number of significantly differentially expressed genes and enriched GO terms than in the associated high concentration treatments. There were also more genes that had increased transcript abundance in both low concentration treatments than upregulated genes across other treatment combinations (Figure 8A). This outcome suggests that grazing defenses may be more important during bloom initiation when *A. anophagefferens* densities are lower and grazing pressure may be higher (Deonarine et al., 2006). At higher cell concentrations, there may be a reservoir of defense chemicals and/or a critical mass of cell density that makes eliciting these defense pathways less important. This would potentially lead to the downregulation of grazing defenses to allocate resources to other processes, such as nutrient acquisition, for example. Another consideration is that ecological interactions can be complex, and *A. anophagefferens* may utilize multiple defense mechanisms and/or chemicals (Poulson et al., 2009) whose upregulation may be density-dependent, further complicating the transcriptional signatures seen in this study.

### Direct vs. indirect exposure

Indirect exposure to planktonic grazers has been shown to elicit a defense response similar to that instigated through direct exposure in other HAB species including *Alexandrium fundyense* (Senft-Batoh et al., 2015). In this study, both direct and indirect exposure to grazers led to distinct changes in gene expression in *A. Anophagefferens.* For example, PCA showed that direct exposure groups clustered together and separate from control treatments as did both indirect exposure groups indicating exposure to grazing filtrate alone elicited a substantial transcriptomic response that differed from direct contact. Direct exposure to grazers generally yielding a greater number of differentially expressed genes and more significantly upregulated KEGG pathways and enriched GO terms. There were also more genes common to both direct exposure treatments with significant decreases in transcript abundance and a relatively large number with increased transcript abundance (Figures 8A,B). Infochemicals related to grazing were likely less persistent and sustained in the indirect exposure treatments given the periodicity of filtrate additions (Van Donk et al., 2011). There were, however, notable changes in gene expression in the indirect exposure treatments, particularly related to the production of sterols and polyketides in the indirect, low treatment. This supports the possibility that chemical signaling cues may be involved in the response of *A. anophagefferens* to grazers. In addition, some pathways were significantly upregulated across all treatments relative to the control, suggesting these were related to persistent, dissolved signaling compounds.

### Toxins and secondary metabolites

Secondary metabolites serve a variety of functions including as toxins and antibiotics and are often important in ecological interactions (Poulson et al., 2009). Multiple HAB species produce secondary metabolites that can inhibit grazing by zooplankton (Kellmann et al., 2010; Penn et al., 2014) and, in some cases, the production of these secondary metabolites is stimulated and upregulated by the presence of grazers (Yang et al., 2011; Senft-Batoh et al., 2015). The KEGG pathway for the biosynthesis of secondary metabolites was significantly upregulated in the direct, high group and marginally upregulated in direct, low. Additionally, several GO terms related to drugs and toxins were enriched, especially in the direct, low treatment. Many of the genes within these pathways and gene sets were related to polyketide and sterol synthesis, and are discussed in detail below.

### Polyketides

Polyketides are a diverse class of bioactive compounds produced by a host of terrestrial, freshwater, and marine organisms that have anti-microbial, anti-fungal, and anti-parasite functionalities (Agrawal et al., 2017). There are dozens of polyketide metabolite families known to be produced by phytoplankton, including all 45 known secondary metabolite families produced by dinoflagellates (Kellmann et al., 2010). Polyketides are synthesized through the iterative addition of extender groups such as acetate, glycolate, malonate, butyrate, and others to an acyl-coenzyme A (CoA) backbone by a type I, II, or III polyketide synthase (PKS) and type I and II PKSs use acyl carrier proteins to activate acyl CoA substrates (Freitag et al., 2011). There were three acyl carrier protein genes with increased transcript abundance in the direct, low treatment and one in the direct, high. Related to acyl carrier proteins, a phosphopantetheine binding gene had increased transcript abundance in the indirect, low treatment. Phosphopentetheine is an essential cofactor in polyketide synthesis that attaches to CoA in acyl carrier proteins (Kleinkauf, 2000). Polyketides can then be further modified, including through oxidation by monooxygenases (Kellmann et al., 2010). Two antibiotic monooxygenase genes had higher transcript abundances in the direct, low treatment.

Polyketide synthases are multi-protein complexes that contain multiple domains including the obligate ketoacylsynthase, acyltransferase, and acyl carrier protein domains along with other optional domains that contribute to the vast structural diversity of polyketides found in nature (Kellmann et al., 2010). In addition to the acyl carrier protein genes mentioned above, an acyltransferase GO term was enriched in both the indirect, high and indirect, low treatments. Kohli et al. (2016) found that the *A. anophagefferens* CCMP strain 1850 transcriptome contains seven polyketide synthase genes and highlighted their potential role in the toxicity of brown tides to zooplankton and bivalves. These genes encode ketoacyl acyl carrier protein synthases and reductases, an enoyl ACP reductase, an ACP S-malonyltransacylase, and a 3-hydroxyacyl-ACP dehydratase (Kohli et al., 2016). A very-long-chain 3-ketoacyl-CoA synthase activity gene had an increase in transcript abundance in the indirect, low treatment. The ketoacylsynthase domain is responsible for forming the common carbon backbone found in many polyketides through successive condensation steps of acetyl CoA or malonyl CoA precursors (Meyer et al., 2015). Multiple genes related to acetyl CoA metabolism had increased transcript abundance in each treatment.

While there were multiple data highlighting activity at different general steps of the polyketide synthesis process, there was also a gene encoding a protein specific to the biosynthesis of the polyketide, fumonisin, that had increased transcript abundance in all treatments except the indirect, high treatment. Fumonisins are known to be highly toxic to a broad array of organisms (Stockmann-Juvala and Savolainen, 2008). Additionally, a macrocin-O-methyltransferase (TylF) gene had lower transcript abundance in both of the low concentration treatments. This gene metabolizes macrocin, a polyketide antibiotic, into tylosin, a related polyketide antibiotic (Seno and Baltz, 1982). The other substrate for this process, in addition to macrocin, is S-adenosyl-L-methionine (SAM) (Seno and Baltz, 1982). SAM acts as a methyl donor in the production of many polyketides (Kellmann et al., 2010). A gene related to SAM transmembrane transport showed significant reduction in transcript abundance across the treatments, potentially implying downregulation of SAM export which would result in increased intracellular concentrations. Another gene encoding the formation of SAM showed increased transcript abundance in all treatments except the indirect, high treatment. Additionally, three GO terms involving SAM were enriched in the direct, high group. Collectively, these findings indicate a broad transcriptional response related to anti-microbial polyketides elicited by exposure to *O. marina*.

Polyketide synthesis is related to fatty acid synthesis and shares similar steps (Kohli et al., 2016). Multiple GO terms with more general annotations referring to fatty acid processes were enriched across all treatments, but particularly in the low concentration treatments. All treatments also had several fatty acid synthesis genes that displayed increased transcript abundance, especially in the low concentration treatments. Jüttner (2001) found that polyunsaturated fatty acids in biofilms produced by diatoms acted as a toxic grazing deterrent to copepods. *Pseudochattonella farcimen* produces polyunsaturated fatty acids that exert toxic effects on grazers (Dittami et al., 2012).

These findings appear to support the hypothesis that *A. anophagefferens* upregulates polyketide synthesis in response to zooplankton. GO analysis indicated that there was increased transcript abundance for gene sets generally related to fatty acid and polyketide synthesis pathways. On a more granular level, a gene for a specific polyketide toxin, fumonisin, had increased transcript abundance in three treatments. Several other genes necessary to produce polyketides including SAM and acetyl CoA metabolism and acyl carrier proteins and their protein domains had increased transcript abundance. Further investigation into the metabolome of this alga could help further understand the role of polyketides as an induced defense response to zooplankton grazers.

### Sterols

Sterols comprise a group of bioactive compounds that can have antibiotic, antiviral, and antioxidant effects (Fagundes and Wagner, 2021). *A. anophagefferens* and other members of Pelagophyceae produce the unique sterol compound 24-propylidenecholesterol (Giner and Boyer, 1998; Giner et al., 2009) and traces of other rare sterols have been detected in *A. anophagefferens* (Giner et al., 2009). The unique composition of sterols found in *A. anophagefferens* may function to inhibit assimilation of dietary sterols in predators, as seen in other HABs (Gobler et al., 2004). Sterols are produced from isoprenoid building blocks of either isopentenyl diphosphate or dimethylallyl pyrophosphate produced by the mevalonic acid (MVA) or the 2-C-methyl-D-erythritol-4-phosphate (MEP) pathways (Fagundes and Wagner, 2021). A dimethylallyltranstransferase gene displayed increased transcript abundance in both of the low treatments and an isopentenyl-diphosphate delta-isomerase gene displayed increased transcript abundance in the direct, low treatment. Also, genes for isoprenoid biosynthesis and 2-C-methyl-D-erythritol 2,4-cyclodiphosphate synthase activity (Kemp et al., 2002) had marginal increases in transcript abundance in the indirect, low treatment. Activity related to steroids was also apparent within a hormone synthesis KEGG pathway that was upregulated in both indirect treatments and a hormone-related GO term enriched in both direct treatments, along with other general sterol terms. Additionally, there were multiple genes related to sterol synthesis and steroids that had increased transcript abundances, particularly in the low concentration treatments. Given these findings, it seems that *A. anophagefferens* upregulates the biosynthesis of some sterols in response to zooplankton, potentially as a defense mechanism. Further investigation into the metabolome of this alga could help advance this hypothesis.

### Polysaccharides

*A. anophagefferens* is coated with extracellular polysaccharides that inhibit bivalve feeding and likely interfere with zooplankton grazing (Sieburth et al., 1988; Gainey and Shumway, 1991). This is thought to be an important feature in its bloom-forming capabilities by reducing mortality losses and allowing blooms to form (Gobler et al., 2004). Multiple GO terms related to carbohydrate synthesis, such as starch biosynthetic process, were enriched in the direct, high treatment. This appears consistent with research conducted by Liu and Buskey (2000), who found that the related brown tide alga, *Aureoumbra lagunensis*, increases EPS production at higher cell densities. GO analysis also indicated enrichment of gene sets related to the extracellular region in all treatments except for indirect, high.

In addition to activity related to general carbohydrate synthesis and the extracellular region, a gene responsible for mannosyl-oligosaccharide 1,2-alpha-mannosidase activity showed a large increase in transcript abundance in the direct, low treatment. This enzyme is involved in the production of glycoproteins (Tulsiani et al., 1982), carbohydrate molecules that have undergone glycosylation and that play a key role in the extracellular matrix (Kwiatkowska and Kwiatkowska-Korczak, 1999). GO terms related to GDP-mannose, a nucleotide-sugar, were enriched in both of the direct exposure treatments. Nucleotide-sugar transporter genes also displayed increased transcript abundance with relatively high log_2_ fold change in both direct exposure treatments. Nucleotide-sugars are often substrates for glycosylation and are important in biogenesis of the extracellular matrix (Gibeaut, 2000).

The alga *A. anophagefferens* possesses unique features within its genome that it uses to metabolize carbohydrates including oligo- and polysaccharide transporter genes that are absent from the genomes of competing phytoplankton species (Gobler et al., 2011). Some of these genes are responsible for D-xylose uptake (Gobler et al., 2011). D-xylose inhibits the hemolytic effects of the toxic glycolipid produced by *A. anophagefferens* in sea bream (Huang et al., 2020) and D-xylose 1-dehydrogenase genes displayed increased transcript abundance in the direct exposure treatments. The upregulation of this gene could provide a protective effect for *A. anophagefferens* as it concurrently produces toxic glycolipids.

### Other putative defenses

Sequencing of the *A. anophagefferens* genome revealed that it contained several genes not present in competing phytoplankton that may be involved in defense against predators including a chloroquine transporter, an erythromycin esterase, a membrane attack complex, multiple berberine bridge enzymes, and phenazine biosynthesis proteins (Gobler et al., 2011). During this study, while transcripts for these genes were found, their abundance in experimental treatments were not significantly different than unexposed controls. While these genes may play a role in predator defense, they were not specifically upregulated by direct or indirect exposure to *O. marina*.

## Conclusion

Zooplankton grazing is a major source of phytoplankton mortality and plays an important role in algal bloom development. *A. anophagefferens* can experience lower grazing mortality compared to other phytoplankton, supporting bloom formation by reducing loss rates. This study demonstrated that during exposure to the microzooplankton grazer, *O. marina*, *A. anophagefferens* experienced smaller loss rates than a non-harmful alga and displayed changes in gene expression that would support the synthesis of multiple classes of bioactive secondary metabolites including polyketides, sterols, and polysaccharides as well as the extracellular matrix. The collective production of these compounds may contribute to grazing deterrence and, thus, advance HAB development. Collectively, these findings provide new insights regarding the ability of HABs to upregulate physiological responses to minimize grazing during blooms.

## Data availability statement

The data presented in this study are deposited in the NCBI repository under accession number PRJNA1018530 (https://www.ncbi.nlm.nih.gov/bioproject/PRJNA1018530).

## Author contributions

WD: Data curation, Formal analysis, Investigation, Methodology, Validation, Visualization, Writing – original draft, Writing – review & editing. CG: Conceptualization, Funding acquisition, Methodology, Project administration, Resources, Supervision, Validation, Writing – original draft, Writing – review & editing.
