# Supplementary material for: Transcription of biochemical defenses by the harmful brown tide pelagophyte, Aureococcus anophagefferens, in response to the protozoan grazer, Oxyrrhis marina
Source: Front Microbiol. 2023 Dec 13;14:1295160. doi: 10.3389/fmicb.2023.1295160 (PMC10756674; doi:10.3389/fmicb.2023.1295160)
Supplement: Supplementary file 1 [file Data_Sheet_1.docx]

**SUPPLEMENTAL FIGURES**


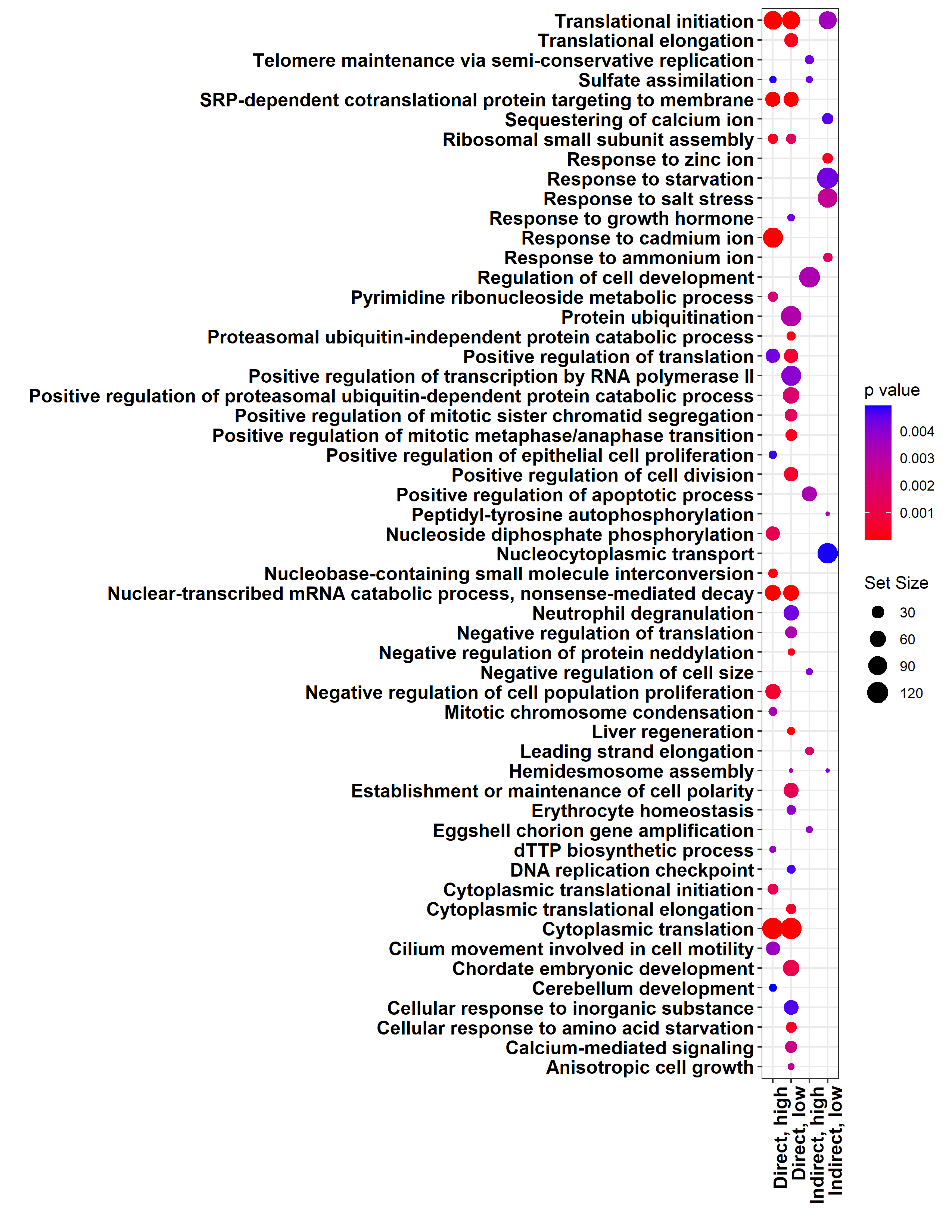


**Supplemental Figure 1**. Significantly enriched GO Biological Processes (BP) (p<0.005). Set size is the total number of genes associated with each gene set.


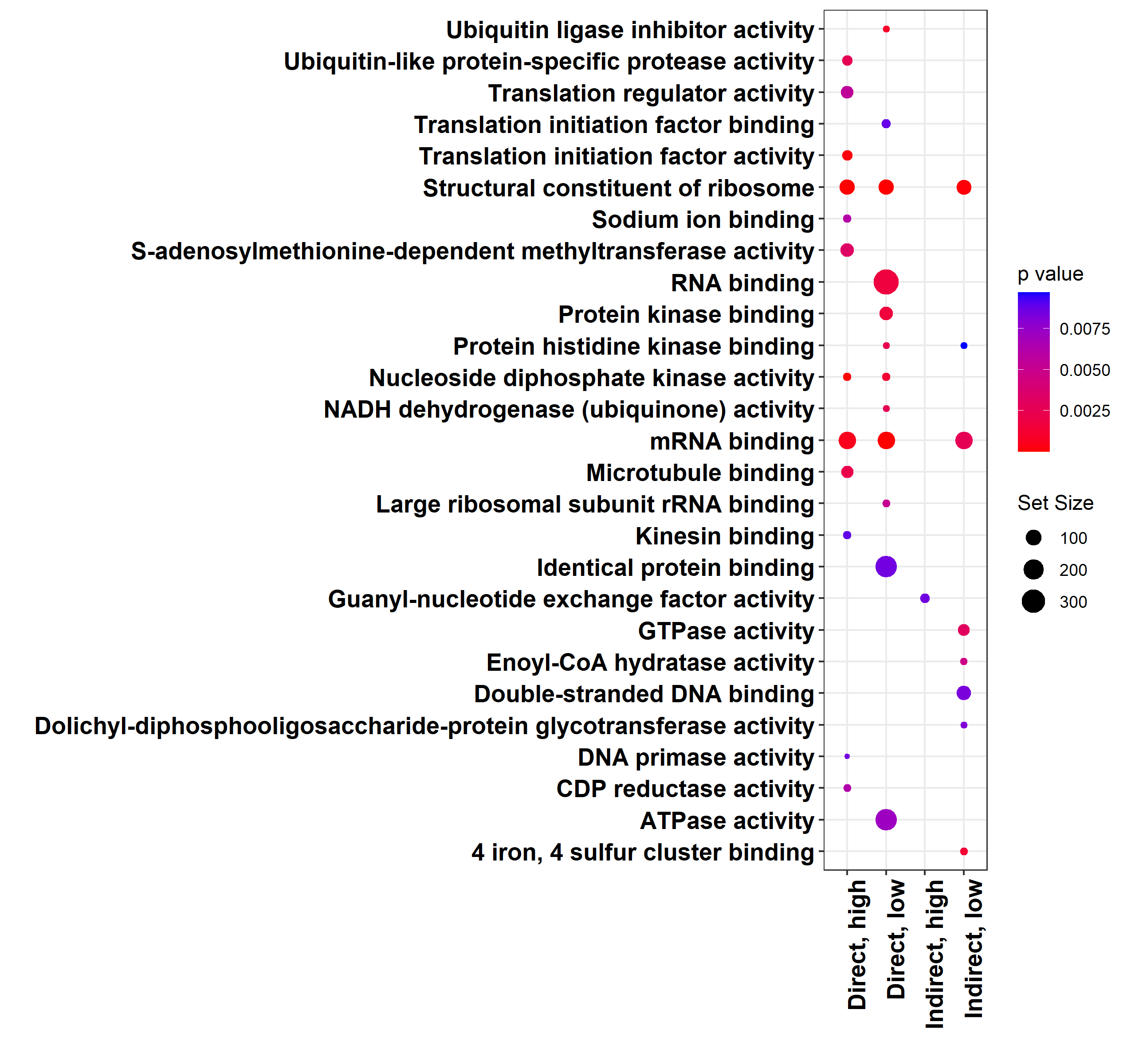


**Supplemental Figure 2**. Significantly enriched GO Molecular Functions (MF) (p<0.01). Set size is the total number of genes associated with each gene set.

**
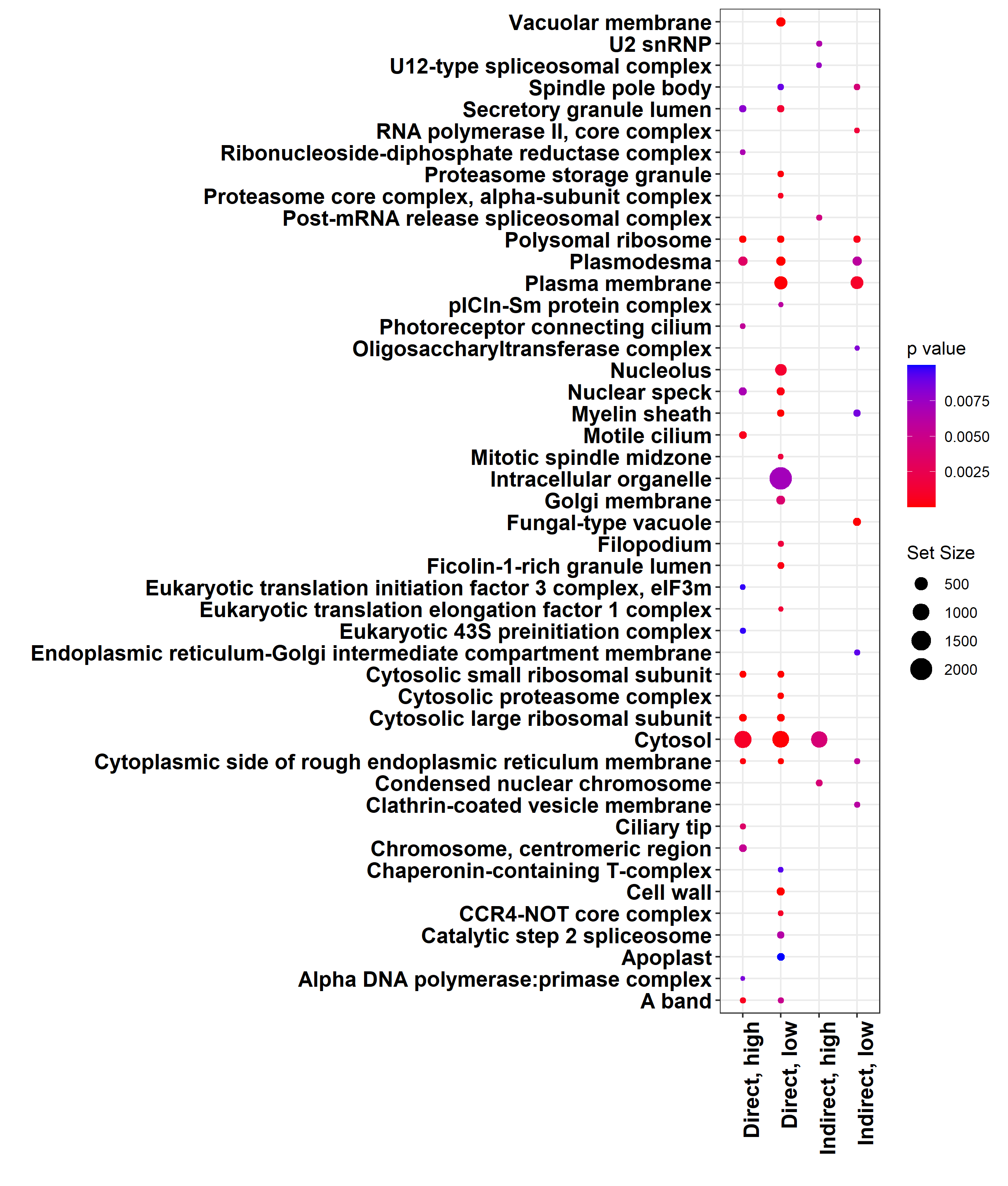
**

**Supplemental Figure 3**. Significantly enriched GO Cellular Components (CC) (p<0.01). Set size is the total number of genes associated with each gene set.

**
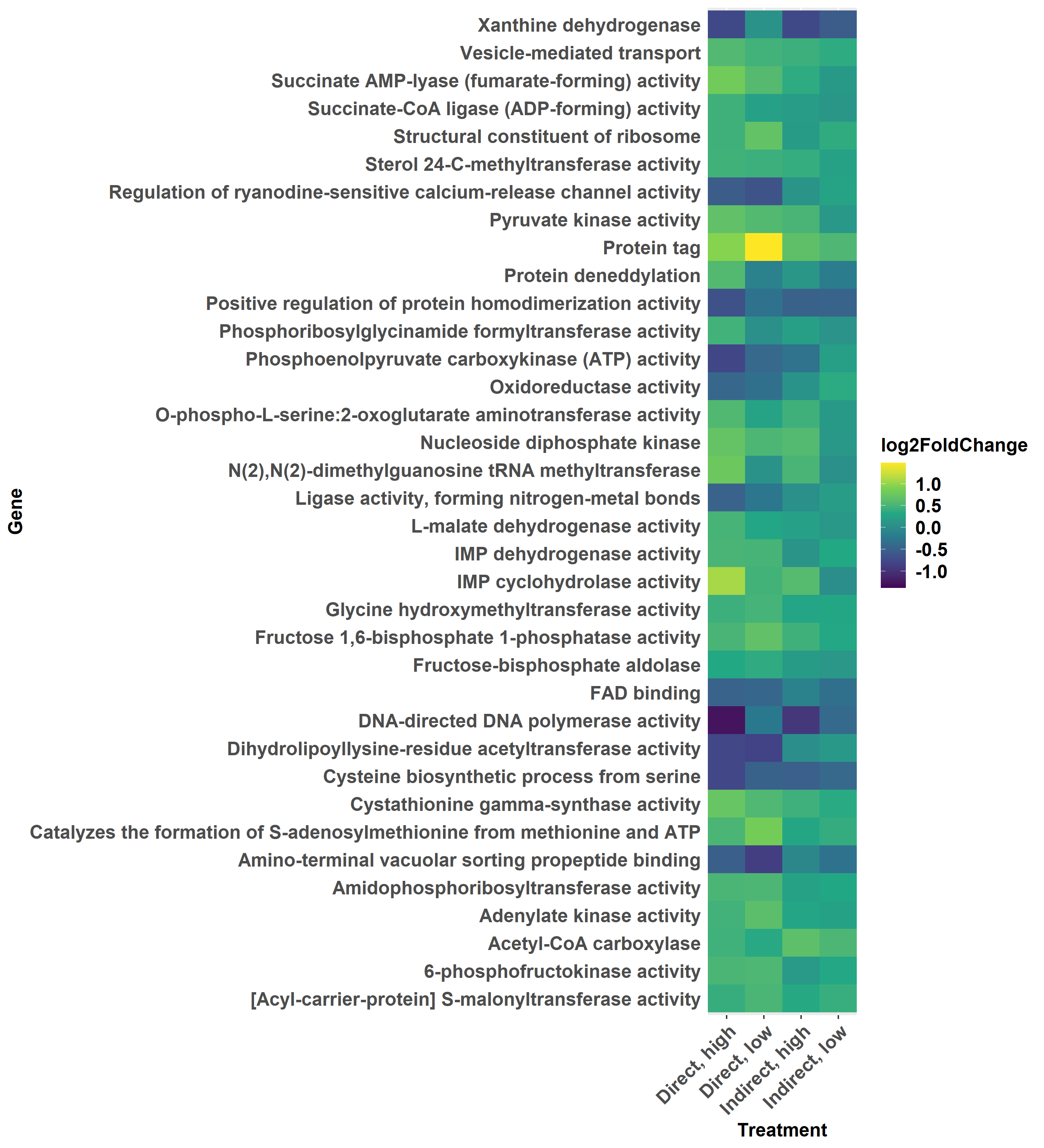
**

**Supplemental Figure 4**. Expression of genes from the secondary metabolite KEGG pathway that were significantly differentially expressed in the direct, high treatment.

**SUPPLEMENTAL TABLES**

| **Sample** | **Paired Alignment Rate (%)** | **Unpaired Forward Alignment Rate (%)** | **Unpaired Reverse Alignment Rate (%)** |
| --- | --- | --- | --- |
| **Direct, low 1** | **42.52** | **46.51** | **52.7** |
| **Direct, low 2** | **28.62** | **32.47** | **38.49** |
| **Direct, low 3** | **35.24** | **38.89** | **40.55** |
| **Direct, high 1** | **61.19** | **63.25** | **67.65** |
| **Direct, high 2** | **60.59** | **62.67** | **67.92** |
| **Direct, high 3** | **60.34** | **62.86** | **68.24** |
| **Indirect, low 1** | **80.6** | **79.48** | **79.68** |
| **Indirect, low 2** | **79.36** | **78.37** | **78.05** |
| **Indirect, low 3** | **81.65** | **80.21** | **80.94** |
| **Indirect, high 1** | **82.96** | **81.23** | **81.95** |
| **Indirect, high 2** | **82.45** | **81.04** | **82.29** |
| **Indirect, high 3** | **80.44** | **80.03** | **80.4** |
| **Control, low 1** | **75.48** | **75.71** | **76.04** |
| **Control, low 2** | **78.51** | **77.95** | **78.04** |
| **Control, low 3** | **78.51** | **77.44** | **77.29** |
| **Control, high 1** | **80.46** | **78.7** | **76.52** |
| **Control, high 2** | **83.03** | **81.12** | **81.28** |
| **Control, high 3** | **80.64** | **79.42** | **79.54** |

Supplemental Table 1. STAR alignment rates to the strain 1850 genome post-trimming.

| Treatment | Subontology | Term | *p* value |
| --- | --- | --- | --- |
| Direct, high | Biological Process | Response to peptide hormone | 0.01529 |
| Direct, high | Biological Process | Antibiotic catabolic process | 0.03772 |
| Direct, high | Biological Process | induction by symbiont of host defense response | 0.03803 |
| Direct, high | Biological Process | adenylate cyclase-activating G protein-coupled receptor signaling pathway | 0.01815 |
| Direct, high | Biological Process | GDP-mannose metabolic process | 0.02006 |
| Direct, high | Biological Process | regulation of smoothened signaling pathway | 0.02865 |
| Direct, high | Biological Process | negative regulation of Ras protein signal transduction | 0.03894 |
| Direct, high | Biological Process | positive regulation of smoothened signaling pathway | 0.04172 |
| Direct, high | Biological Process | sucrose biosynthetic process | 0.0423 |
| Direct, high | Biological Process | response to growth hormone | 0.03939 |
| Direct, high | Biological Process | starch biosynthetic process | 0.0431 |
| Direct, high | Biological Process | cellular response to fatty acid | 0.04384 |
| Direct, high | Biological Process | gluconeogenesis | 0.04424 |
| Direct, high | Biological Process | hormone-mediated signaling pathway | 0.04643 |
| Direct, high | Biological Process | S-adenosylmethionine cycle | 0.04379 |
| Direct, high | Molecular Function | S-adenosylmethionine-dependent methyltransferase activity | 0.00324 |
| Direct, high | Molecular Function | [acyl-carrier-protein] S-malonyltransferase activity | 0.04722 |
| Direct, high | Molecular Function | Sterol 14-demethylase activity | 0.07808 |
| Direct, high | Molecular Function | Toxic substance binding | 0.09719 |
| Direct, high | Cellular Component | cell wall | 0.01289 |
| Direct, high | Cellular Component | hyphal cell wall | 0.04 |
| Direct, low | Biological Process | Hemidesmosome assembly | 0.00339 |
| Direct, low | Biological Process | response to growth hormone | 0.00432 |
| Direct, low | Biological Process | Cellular response to antibiotic | 0.01105 |
| Direct, low | Biological Process | Sterol regulatory element binding protein cleavage | 0.01206 |
| Direct, low | Biological Process | Toxin transport | 0.01929 |
| Direct, low | Biological Process | Cellular response to toxic substance | 0.01993 |
| Direct, low | Biological Process | cellular response to drug | 0.03133 |
| Direct, low | Biological Process | Regulation of response to drug | 0.03882 |
| Direct, low | Biological Process | cell adhesion | 0.03839 |
| Direct, low | Biological Process | fatty acid elongation, saturated fatty acid | 0.04493 |
| Direct, low | Biological Process | fatty acid elongation, monounsaturated fatty acid | 0.04493 |
| Direct, low | Biological Process | fatty acid elongation, polyunsaturated fatty acid | 0.04493 |
| Direct, low | Molecular Function | fatty acid elongase activity | 0.04605 |
| Direct, low | Molecular Function | Cyclosporin A binding | 0.03169 |
| Direct, low | Molecular Function | GDP-mannose 3,5-epimerase activity | 0.03365 |
| Direct, low | Molecular Function | Drug binding | 0.06436 |
| Direct, low | Cellular Component | Hemidesmosome | 0.02276 |
| Direct, low | Cellular Component | Extracellular matrix | 0.02367 |
| Direct, low | Cellular Component | cell wall | 3E-08 |
| Direct, low | Cellular Component | extracellular region | 0.02584 |
| Indirect, high | Biological Process | Antibiotic metabolic process | 0.0275 |
| Indirect, high | Biological Process | (17Z)-protosta-17(20),24-dien-3beta-ol biosynthetic process | 0.0272 |
| Indirect, high | Biological Process | neuropeptide signaling pathway | 0.0481 |
| Indirect, high | Biological Process | positive regulation of choline O-acetyltransferase activity | 0.0481 |
| Indirect, high | Biological Process | positive regulation of insulin receptor signaling pathway | 0.0463 |
| Indirect, high | Biological Process | positive regulation of fatty acid oxidation | 0.0359 |
| Indirect, high | Biological Process | phosphatidylinositol phosphorylation | 0.0237 |
| Indirect, high | Molecular Function | Lanosterol synthase activity | 0.0276 |
| Indirect, high | Molecular Function | diacylglycerol O-acyltransferase activity | 0.0405 |
| Indirect, high | Molecular Function | fatty acid binding | 0.0423 |
| Indirect, high | Molecular Function | Sterol 14-demethylase activity | 0.0439 |
| Indirect, low | Biological Process | Hemidesmosome assembly | 0.00428 |
| Indirect, low | Biological Process | Drug metabolic process | 0.01129 |
| Indirect, low | Biological Process | Pseudohyphal growth | 0.02301 |
| Indirect, low | Biological Process | Cellular response to toxic substance | 0.02713 |
| Indirect, low | Biological Process | Regulation of synaptic transmission, glutamatergic | 0.0294 |
| Indirect, low | Biological Process | Cellular response to antibiotic | 0.04388 |
| Indirect, low | Biological Process | Regulation of signaling receptor activity | 0.04849 |
| Indirect, low | Biological Process | positive regulation of glucocorticoid receptor signaling pathway | 0.03274 |
| Indirect, low | Molecular Function | dolichyl-diphosphooligosaccharide-protein glycotransferase activity | 0.00806 |
| Indirect, low | Molecular Function | acetyl-CoA C-acyltransferase activity | 0.03093 |
| Indirect, low | Cellular Component | oligosaccharyltransferase complex | 0.0083 |
| Indirect, low | Cellular Component | cell wall | 0.0151 |
| Indirect, low | Cellular Component | hemidesmosome | 0.0264 |
| Indirect, low | Cellular Component | cell periphery | 0.0254 |

Supplemental Table 2. Enriched gene Ontology (GO) terms potentially related to a defense response.

| Treatment | Query | Description | log2FoldChange | padj |
| --- | --- | --- | --- | --- |
| Direct, high | JL721_4977 | holo-[acyl-carrier-protein] synthase activity | -1.565129079 | 0.000326328 |
| Direct, high | JL721_8672 | transferase activity, transferring acyl groups | -1.822969668 | 0.027497745 |
| Direct, high | JL721_4766 | [acyl-carrier-protein] S-malonyltransferase activity | 0.410451381 | 0.016799915 |
| Direct, high | JL721_9309 | Fatty acid desaturase | 0.529716234 | 0.006646887 |
| Direct, high | JL721_837 | S-adenosyl-L-methionine transmembrane transport | -1.15566685 | 5.60E-06 |
| Direct, high | JL721_6224 | Catalyzes the formation of S-adenosylmethionine from methionine and ATP | 0.519097614 | 0.00656958 |
| Direct, high | JL721_7632 | acetyl-CoA carboxylase | -1.003085849 | 3.98E-07 |
| Direct, high | JL721_2090 | acetyl-CoA carboxylase | 0.658804046 | 0.000137371 |
| Direct, high | JL721_12961 | acetyl-CoA carboxylase | 0.456024779 | 0.01276562 |
| Direct, high | JL721_9923 | pyruvate dehydrogenase (acetyl-transferring) kinase activity | -2.552612594 | 0.027981782 |
| Direct, high | JL721_2862 | S-adenosyl-L-methionine transmembrane transport | -1.512808073 | 0.024095148 |
| Direct, high | JL721_10524 | Protein involved in biosynthesis of mitomycin antibiotics polyketide fumonisin | 0.516338204 | 0.031046945 |
| Indirect, high | JL721_837 | S-adenosyl-L-methionine transmembrane transport | -0.758024519 | 0.01335499 |
| Indirect, high | JL721_2090 | acetyl-CoA carboxylase | 0.837438321 | 2.98E-07 |
| Indirect, high | JL721_12961 | acetyl-CoA carboxylase | 0.652188269 | 0.000413965 |
| Direct, low | JL721_8187 | [acyl-carrier-protein] S-malonyltransferase activity | 0.928802582 | 0.000239653 |
| Direct, low | JL721_11196 | long-chain-3-hydroxyacyl-CoA dehydrogenase activity | 0.46704504 | 0.014479425 |
| Direct, low | JL721_2055 | [acyl-carrier-protein] S-malonyltransferase activity | -0.81492307 | 0.017349371 |
| Direct, low | JL721_4766 | [acyl-carrier-protein] S-malonyltransferase activity | 0.51596749 | 0.019985009 |
| Direct, low | JL721_7344 | transferase activity, transferring acyl groups | -3.450644079 | 0.034051569 |
| Direct, low | JL721_9812 | 3-oxoacyl-[acyl-carrier-protein] synthase activity | -0.782248582 | 0.041041595 |
| Direct, low | JL721_4890 | fatty acid elongation, saturated fatty acid | 1.116377773 | 4.37E-16 |
| Direct, low | JL721_9309 | Fatty acid desaturase | 0.866743712 | 4.10E-07 |
| Direct, low | JL721_5325 | unsaturated fatty acid biosynthetic process | 0.875079851 | 8.36E-07 |
| Direct, low | JL721_11506 | unsaturated fatty acid biosynthetic process | 0.58663153 | 4.38E-05 |
| Direct, low | JL721_5312 | fatty acid elongation, saturated fatty acid | 0.734490017 | 0.002284634 |
| Direct, low | JL721_10751 | fatty acid elongation, saturated fatty acid | 0.577524813 | 0.01295829 |
| Direct, low | JL721_10939 | palmitoyl-(protein) hydrolase activity | -1.298338627 | 9.22E-08 |
| Direct, low | JL721_3753 | palmitoyl-(protein) hydrolase activity | 0.792001634 | 0.000293057 |
| Direct, low | JL721_11201 | phosphopantetheine binding | -1.774296893 | 0.025242662 |
| Direct, low | JL721_4843 | phosphopantetheine binding | 4.941173049 | NA |
| Direct, low | JL721_10199 | 1-acylglycerol-3-phosphate O-acyltransferase activity | -1.096392462 | 0.00181391 |
| Direct, low | JL721_837 | S-adenosyl-L-methionine transmembrane transport | -1.700279492 | 1.69E-17 |
| Direct, low | JL721_1454 | Polyketide synthase modules and related proteins | 0.328569617 | 0.096951775 |
| Direct, low | JL721_7369 | Macrocin-O-methyltransferase (TylF) | -1.47401228 | 0.080130037 |
| Direct, low | JL721_10524 | Protein involved in biosynthesis of mitomycin antibiotics polyketide fumonisin | 0.389824318 | 0.060250265 |
| Direct, low | JL721_6224 | Catalyzes the formation of S-adenosylmethionine from methionine and ATP | 0.842158275 | 1.86E-13 |
| Direct, low | JL721_11226 | 3-oxo-behenoyl-CoA reductase activity | 0.856307151 | 0.00033012 |
| Direct, low | JL721_7632 | acetyl-CoA carboxylase | -0.998805026 | 5.23E-09 |
| Direct, low | JL721_5685 | pyruvate dehydrogenase (acetyl-transferring) activity | 0.646357545 | 5.86E-06 |
| Direct, low | JL721_2091 | acetyl-CoA carboxylase | 0.517195934 | 0.001223182 |
| Direct, low | JL721_12961 | acetyl-CoA carboxylase | 0.351135188 | 0.038861725 |
| Direct, low | JL721_705 | protein deacetylase activity | -1.454850045 | 0.048096099 |
| Direct, low | JL721_3728 | acetylation-dependent protein binding | -0.487684451 | 0.049010371 |
| Direct, low | JL721_2332 | Antibiotic biosynthesis monooxygenase | 0.608149149 | 0.000164219 |
| Direct, low | JL721_3539 | Antibiotic biosynthesis monooxygenase | 0.788820812 | 0.008070547 |
| Indirect, low | JL721_11196 | long-chain-3-hydroxyacyl-CoA dehydrogenase activity | 0.450338277 | 0.02244499 |
| Indirect, low | JL721_12780 | long-chain-3-hydroxyacyl-CoA dehydrogenase activity | 0.457969209 | 0.038221054 |
| Indirect, low | JL721_8712 | very-long-chain 3-ketoacyl-CoA synthase activity | 0.963604239 | 0.039637598 |
| Indirect, low | JL721_4890 | fatty acid elongation, saturated fatty acid | 0.8031137 | 4.77E-08 |
| Indirect, low | JL721_9309 | Fatty acid desaturase | 0.721385753 | 0.000114566 |
| Indirect, low | JL721_3554 | fatty acid biosynthetic process | 0.427330725 | 0.008192937 |
| Indirect, low | JL721_5325 | unsaturated fatty acid biosynthetic process | 0.452943354 | 0.041445784 |
| Indirect, low | JL721_11506 | unsaturated fatty acid biosynthetic process | 0.35274654 | 0.043510878 |
| Indirect, low | JL721_10893 | protein-cysteine S-palmitoyltransferase activity | 0.591078777 | 0.033717793 |
| Indirect, low | JL721_1146 | protein-cysteine S-palmitoyltransferase activity | -0.624040377 | 0.049249296 |
| Indirect, low | JL721_3913 | phosphopantetheine binding | 0.451701022 | 0.003409725 |
| Indirect, low | JL721_7369 | Macrocin-O-methyltransferase (TylF) | -1.708645716 | 0.034131689 |
| Indirect, low | JL721_10524 | Protein involved in biosynthesis of mitomycin antibiotics polyketide fumonisin | 0.37501562 | 0.081190987 |
| Indirect, low | JL721_2332 | Antibiotic biosynthesis monooxygenase | 0.342086612 | 0.081054803 |
| Indirect, low | JL721_6224 | Catalyzes the formation of S-adenosylmethionine from methionine and ATP | 0.397603996 | 0.0043 |
| Indirect, low | JL721_2091 | acetyl-CoA carboxylase | 0.586411937 | 0.0002 |
| Indirect, low | JL721_12961 | acetyl-CoA carboxylase | 0.529529452 | 0.0012 |
| Indirect, low | JL721_5685 | pyruvate dehydrogenase (acetyl-transferring) activity | 0.503535657 | 0.0015 |
| Indirect, low | JL721_2090 | acetyl-CoA carboxylase | 0.581631493 | 0.0472 |
| Indirect, low | JL721_837 | S-adenosyl-L-methionine transmembrane transport | -1.014594621 | 2.25E-08 |

Supplemental Table 3. Putative polyketide and fatty acid synthesis genes.

| Treatment | Description | *p* Value |
| --- | --- | --- |
| Direct, high | ko01110 Biosynthesis of secondary metabolites | 0.000620348 |
| Direct, high | ko01240 Biosynthesis of cofactors | 0.006333772 |
| Direct, high | ko00983 Drug metabolism - other enzymes | 0.07078437 |
| Direct, high | ko00770 Pantothenate and CoA biosynthesis | 0.081279818 |
| Direct, high | ko00520 Amino sugar and nucleotide sugar metabolism | 0.097944529 |
| Indirect, high | ko04927 Cortisol synthesis and secretion | 0.054167334 |
| Indirect, high | ko04925 Aldosterone synthesis and secretion | 0.067728546 |
| Indirect, high | ko00513 Various types of N-glycan biosynthesis | 0.079464467 |
| Direct, low | ko01240 Biosynthesis of cofactors | 0.059448832 |
| Direct, low | ko01110 Biosynthesis of secondary metabolites | 0.062574707 |
| Direct, low | ko00770 Pantothenate and CoA biosynthesis | 0.097928396 |
| Indirect, low | ko04927 Cortisol synthesis and secretion | 0.034521897 |
| Indirect, low | ko04925 Aldosterone synthesis and secretion | 0.060249766 |

Supplemental Table 4. Upregulated KEGG pathways potentially related to polyketide and sterol synthesis.

| Treatment | Query | Description | log2FoldChange | padj |
| --- | --- | --- | --- | --- |
| Direct, high | JL721_6963 | sterol 24-C-methyltransferase activity | 0.458141455 | 0.00281914 |
| Direct, high | JL721_12480 | sterol 24-C-methyltransferase activity | 0.333958653 | 0.057228496 |
| Direct, high | JL721_10587 | sterol 24-C-methyltransferase activity | 0.458675989 | 0.080030159 |
| Direct, high | JL721_4141 | 13-prostaglandin reductase activity | -2.597235019 | 0.093675519 |
| Direct, high | JL721_5085 | C-4 methylsterol oxidase activity | -4.730787513 | NA |
| Indirect, high | JL721_4065 | sterol 14-demethylase activity | 0.510634534 | 0.003269646 |
| Indirect, high | JL721_6963 | sterol 24-C-methyltransferase activity | 0.407856593 | 0.024094889 |
| Indirect, high | JL721_10587 | sterol 24-C-methyltransferase activity | 0.565256144 | 0.045985681 |
| Direct, low | JL721_4065 | sterol 14-demethylase activity | 0.885175236 | 7.59E-06 |
| Direct, low | JL721_6963 | sterol 24-C-methyltransferase activity | 0.437098221 | 0.001035432 |
| Direct, low | JL721_5244 | Belongs to the sterol desaturase family | 0.60295752 | 0.00190902 |
| Direct, low | JL721_10587 | sterol 24-C-methyltransferase activity | 0.716915301 | 0.011978757 |
| Direct, low | JL721_5756 | isopentenyl-diphosphate delta-isomerase activity | 0.443102639 | 0.029335658 |
| Direct, low | JL721_12311 | 3-beta-hydroxy-delta5-steroid dehydrogenase activity | 0.81732725 | 1.52E-08 |
| Direct, low | JL721_1110 | prostaglandin-F synthase activity | 0.402474962 | 0.037939046 |
| Direct, low | JL721_3396 | isoprenoid biosynthetic process | -0.600535358 | 0.04063955 |
| Direct, low | JL721_7100 | Isoprenylcysteine carboxyl methyltransferase (ICMT) family | 0.834650144 | 0.039891447 |
| Direct, low | JL721_4942 | dimethylallyltranstransferase activity | 0.532658839 | 0.008109231 |
| Indirect, low | JL721_4942 | dimethylallyltranstransferase activity | 0.445976478 | 0.049996386 |
| Indirect, low | JL721_4065 | sterol 14-demethylase activity | 1.100050263 | 2.13E-08 |
| Indirect, low | JL721_5244 | Belongs to the sterol desaturase family | 0.458162891 | 0.038871113 |
| Indirect, low | JL721_7011 | 2-C-methyl-D-erythritol 2,4-cyclodiphosphate synthase activity | 0.60438634 | 0.070052795 |
| Indirect, low | JL721_12311 | 3-beta-hydroxy-delta5-steroid dehydrogenase activity | 0.387302531 | 0.027280872 |
| Indirect, low | JL721_10017 | negative regulation of brassinosteroid mediated signaling pathway | 0.383321051 | 0.064947772 |
| Indirect, low | JL721_12358 | isoprenoid biosynthetic process | 0.262072011 | 0.097584515 |

Supplemental Table 5. Putative sterol synthesis genes.

| Treatment | Query | Description | log2FoldChange | padj |
| --- | --- | --- | --- | --- |
| Direct, high | JL721_3592 | D-xylose 1-dehydrogenase (NADP+) activity | 0.524445197 | 0.002969301 |
| Direct, high | JL721_8701 | fructose 1,6-bisphosphate 1-phosphatase activity | 0.511364462 | 0.00562536 |
| Direct, high | JL721_10810 | UDP-galactose transmembrane transporter activity | 0.368029823 | 0.027568865 |
| Direct, high | JL721_12473 | fructose-bisphosphate aldolase | 0.33545589 | 0.039271935 |
| Direct, high | JL721_460 | glucose-6-phosphate isomerase activity | 0.462257205 | 0.045444455 |
| Direct, high | JL721_11315 | carbohydrate transport | -1.200931752 | 3.98E-07 |
| Direct, high | JL721_1144 | carbohydrate transport | 0.548375359 | 0.000419572 |
| Direct, high | JL721_1276 | (1->6)-beta-D-glucan biosynthetic process | -0.83588615 | 0.042881976 |
| Direct, high | JL721_5715 | Phosphoglucomutase/phosphomannomutase, alpha/beta/alpha domain III | -4.587923758 | 0.022728902 |
| Direct, high | JL721_12422 | phosphoacetylglucosamine mutase activity | -1.834537546 | 0.020217476 |
| Direct, high | JL721_9762 | Glucosamine-6-phosphate isomerases/6-phosphogluconolactonase | 0.413516768 | 0.047688969 |
| Direct, high | JL721_7195 | phosphoglycerate kinase activity | -0.494710128 | 0.047356676 |
| Direct, high | JL721_7942 | nucleotide-sugar transmembrane transporter activity | 1.000053485 | 0.007826462 |
| Indirect, high | JL721_10810 | UDP-galactose transmembrane transporter activity | 0.412169356 | 0.024094889 |
| Indirect, high | JL721_8701 | fructose 1,6-bisphosphate 1-phosphatase activity | 0.455501405 | 0.041059115 |
| Indirect, high | JL721_1144 | carbohydrate transport | 0.495124857 | 0.007128706 |
| Indirect, high | JL721_12422 | phosphoacetylglucosamine mutase activity | -1.716451646 | 0.045985681 |
| Direct, low | JL721_1444 | dTDP-glucose 4,6-dehydratase activity | 0.796948042 | 7.71E-10 |
| Direct, low | JL721_8701 | fructose 1,6-bisphosphate 1-phosphatase activity | 0.674887713 | 3.04E-09 |
| Direct, low | JL721_8648 | GDP-mannose 4,6-dehydratase activity | 0.89459102 | 1.83E-06 |
| Direct, low | JL721_3974 | GDP-L-fucose synthase activity | 0.705332466 | 1.54E-05 |
| Direct, low | JL721_8645 | UDP-glucose 6-dehydrogenase activity | 0.499167496 | 0.000136891 |
| Direct, low | JL721_9152 | UDP-glucose:hexose-1-phosphate uridylyltransferase activity | -0.755289112 | 0.000184365 |
| Direct, low | JL721_12473 | fructose-bisphosphate aldolase | 0.379452547 | 0.000488009 |
| Direct, low | JL721_11655 | regulation of pentose-phosphate shunt | 0.378355111 | 0.001973503 |
| Direct, low | JL721_11673 | D-xylose 1-dehydrogenase (NADP+) activity | 0.707817512 | 0.002779366 |
| Direct, low | JL721_12307 | regulation of pentose-phosphate shunt | 0.519240836 | 0.008083684 |
| Direct, low | JL721_9662 | regulation of pentose-phosphate shunt | -1.126449066 | 0.008939438 |
| Direct, low | JL721_6709 | regulation of pentose-phosphate shunt | 1.034125257 | 0.009098229 |
| Direct, low | JL721_11255 | regulation of pentose-phosphate shunt | 0.910256109 | 0.019248183 |
| Direct, low | JL721_1796 | glucose-6-phosphate 1-epimerase activity | 0.482912653 | 0.02296386 |
| Direct, low | JL721_9691 | regulation of pentose-phosphate shunt | 0.56582487 | 0.027819912 |
| Direct, low | JL721_4914 | UDP-galactose transmembrane transporter activity | 0.379977541 | 0.029873801 |
| Direct, low | JL721_1448 | D-xylose 1-dehydrogenase (NADP+) activity | 0.373211122 | 0.034441222 |
| Direct, low | JL721_12186 | Xylose isomerase-like TIM barrel | -1.379208735 | 0.040113578 |
| Direct, low | JL721_9833 | dTDP-glucose 4,6-dehydratase activity | 0.396313948 | 0.046411377 |
| Direct, low | JL721_10360 | mannosyl-oligosaccharide 1,2-alpha-mannosidase activity | 2.455716757 | 0.00818546 |
| Direct, low | JL721_13068 | carbohydrate transport | 0.727303571 | 4.20E-05 |
| Direct, low | JL721_11315 | carbohydrate transport | -1.059527182 | 0.000222221 |
| Direct, low | JL721_11676 | carbohydrate transport | 0.468288509 | 0.000291534 |
| Direct, low | JL721_4428 | racemase and epimerase activity, acting on carbohydrates and derivatives | 0.687935893 | 0.000718316 |
| Direct, low | JL721_8448 | racemase and epimerase activity, acting on carbohydrates and derivatives | 0.578147724 | 0.001073279 |
| Direct, low | JL721_11813 | carbohydrate transmembrane transporter activity | 0.62373169 | 0.008904019 |
| Direct, low | JL721_3154 | racemase and epimerase activity, acting on carbohydrates and derivatives | 0.657812809 | 0.009638385 |
| Direct, low | JL721_2097 | carbohydrate transport | -1.065452465 | 0.017760911 |
| Direct, low | JL721_1144 | carbohydrate transport | 0.358862409 | 0.037717341 |
| Direct, low | JL721_8745 | UDP-glucoronosyl and UDP-glucosyl transferase | -1.889798984 | 0.044423946 |
| Direct, low | JL721_12206 | Concanavalin A-like lectin/glucanases superfamily | -1.634776879 | 0.012781297 |
| Direct, low | JL721_9762 | Glucosamine-6-phosphate isomerases/6-phosphogluconolactonase | 0.744367606 | 1.03E-07 |
| Direct, low | JL721_13142 | alpha-N-acetylglucosaminidase activity | -1.511305234 | 0.000977587 |
| Direct, low | JL721_9939 | alpha-N-acetylglucosaminidase activity | -1.167798598 | 0.001764792 |
| Direct, low | JL721_5109 | protein N-acetylglucosaminyltransferase activity | -0.660794407 | 0.012879984 |
| Direct, low | JL721_6539 | dolichyl pyrophosphate Man9GlcNAc2 alpha-1,3-glucosyltransferase activity | -1.030173981 | 0.01936992 |
| Direct, low | JL721_12422 | phosphoacetylglucosamine mutase activity | -2.708439593 | 0.019877946 |
| Direct, low | JL721_5309 | alpha-1,3-glucosidase activity | -1.721312174 | 0.031013732 |
| Direct, low | JL721_3166 | phosphatidylinositol N-acetylglucosaminyltransferase activity | 0.698608971 | 0.040924733 |
| Direct, low | JL721_8745 | UDP-glucoronosyl and UDP-glucosyl transferase | -1.889798984 | 0.044423946 |
| Direct, low | JL721_621 | pectate lyase activity | 0.339707891 | 0.021946756 |
| Direct, low | JL721_12404 | glyceraldehyde-3-phosphate dehydrogenase (NAD(P)+) (phosphorylating) activity | -0.652960728 | 1.66E-06 |
| Direct, low | JL721_7195 | phosphoglycerate kinase activity | -0.455466437 | 1.50E-05 |
| Direct, low | JL721_9211 | digalactosyldiacylglycerol synthase activity | -0.925152311 | 0.000645736 |
| Direct, low | JL721_10199 | 1-acylglycerol-3-phosphate O-acyltransferase activity | -1.096392462 | 0.00181391 |
| Direct, low | JL721_6750 | glycerol-3-phosphate catabolic process | -1.925796224 | 0.002656568 |
| Direct, low | JL721_6064 | phosphoglycerate dehydrogenase activity | 0.52111328 | 0.005902877 |
| Direct, low | JL721_8595 | phosphoglycerate dehydrogenase activity | 0.472079839 | 0.006971077 |
| Direct, low | JL721_1570 | 1,2-diacylglycerol 3-beta-galactosyltransferase activity | -0.454990977 | 0.026134019 |
| Direct, low | JL721_3803 | glycerol kinase activity | -1.962188624 | 0.046475997 |
| Direct, low | JL721_7851 | pyrimidine nucleotide-sugar transmembrane transporter activity | -1.954040612 | 0.001157774 |
| Direct, low | JL721_2208 | Nucleotide-diphospho-sugar transferase | 0.708752709 | 0.006924867 |
| Direct, low | JL721_4650 | pyrimidine nucleotide-sugar transmembrane transporter activity | 0.958060751 | 0.011965722 |
| Direct, low | JL721_6092 | chitin catabolic process | -1.116512567 | 0.051392162 |
| Direct, low | JL721_4043 | sphingolipid transporter activity | 0.778669689 | 0.051402834 |
| Direct, low | JL721_8518 | heparanase activity | -1.146478134 | 0.056635319 |
| Direct, low | JL721_6836 | mannose-6-phosphate isomerase activity | -1.29169702 | 0.05555915 |
| Direct, low | JL721_9113 | mannose metabolic process | -0.338957344 | 0.092682974 |
| Direct, low | JL721_2776 | COG3306 Glycosyltransferase involved in LPS biosynthesis | -0.647428603 | 0.035515127 |
| Indirect, low | JL721_1444 | dTDP-glucose 4,6-dehydratase activity | 0.427052666 | 0.005270749 |
| Indirect, low | JL721_3974 | GDP-L-fucose synthase activity | 0.487451689 | 0.00778784 |
| Indirect, low | JL721_8976 | UDP-galactose transmembrane transporter activity | 0.589538894 | 0.009173889 |
| Indirect, low | JL721_11590 | sedoheptulose-7-phosphate:D-glyceraldehyde-3-phosphate glyceronetransferase activity | -0.873039607 | 0.015681553 |
| Indirect, low | JL721_8701 | fructose 1,6-bisphosphate 1-phosphatase activity | 0.324040425 | 0.020642737 |
| Indirect, low | JL721_8648 | GDP-mannose 4,6-dehydratase activity | 0.503306075 | 0.024640405 |
| Indirect, low | JL721_11655 | regulation of pentose-phosphate shunt | 0.280464728 | 0.042931589 |
| Indirect, low | JL721_4410 | D-xylose 1-dehydrogenase (NADP+) activity | -0.507480669 | 0.043266307 |
| Indirect, low | JL721_4914 | UDP-galactose transmembrane transporter activity | 0.374204755 | 0.047650719 |
| Indirect, low | JL721_8193 | oligosaccharyl transferase activity | 0.629813956 | 0.007829338 |
| Indirect, low | JL721_11115 | Oligosaccharyl transferase STT3 subunit | 0.448480739 | 0.049148464 |
| Indirect, low | JL721_13068 | carbohydrate transport | 0.906038432 | 1.07E-07 |
| Indirect, low | JL721_1144 | carbohydrate transport | 0.574423536 | 0.000392308 |
| Indirect, low | JL721_11676 | carbohydrate transport | 0.405848523 | 0.003573711 |
| Indirect, low | JL721_3154 | racemase and epimerase activity, acting on carbohydrates and derivatives | 0.659552402 | 0.008830147 |
| Indirect, low | JL721_2939 | carbohydrate binding | -0.437579255 | 0.017426462 |
| Indirect, low | JL721_4626 | carbohydrate transport | 0.480330349 | 0.032314862 |
| Indirect, low | JL721_9762 | Glucosamine-6-phosphate isomerases/6-phosphogluconolactonase | 0.632986978 | 2.39E-05 |
| Indirect, low | JL721_13142 | alpha-N-acetylglucosaminidase activity | -1.223233139 | 0.003017192 |
| Indirect, low | JL721_5109 | protein N-acetylglucosaminyltransferase activity | -0.57451223 | 0.033090033 |
| Indirect, low | JL721_1717 | transferase activity, transferring hexosyl groups | 3.286792945 | NA |
| Indirect, low | JL721_6750 | glycerol-3-phosphate catabolic process | -1.451946393 | 0.011846733 |
| Indirect, low | JL721_4494 | COG0111 Phosphoglycerate dehydrogenase and related dehydrogenases | 0.413465224 | 0.017239335 |
| Indirect, low | JL721_9099 | glycerone kinase activity | -1.29503266 | 0.033717793 |
| Indirect, low | JL721_9211 | digalactosyldiacylglycerol synthase activity | -0.584280246 | 0.044651719 |
| Indirect, low | JL721_2208 | Nucleotide-diphospho-sugar transferase | 0.694681106 | 0.008192937 |

Supplemental Table 6. Carbohydrate metabolism and transport genes.

| Treatment | Query | Description | log2FoldChange | padj |
| --- | --- | --- | --- | --- |
| Direct, high | JL721_11208 | sulfide:quinone oxidoreductase activity | 0.655506854 | 0.01159201 |
| Direct, high | NA | indole-3-glycerol-phosphate synthase activity | -1.097857476 | 0.10629459 |
| Direct, high | JL721_11702 | Putative small multi-drug export protein | 0.828012353 | 0.10059471 |
| Direct, low | JL721_2978 | cytolysis | -0.789438882 | 0.00501362 |
| Direct, low | JL721_2979 | cytolysis | -1.518719921 | 0.09235648 |
| Direct, low | JL721_7974 | toxin transport | -0.682990363 | 0.01204788 |
| Direct, low | JL721_4367 | cellular response to paclitaxel | 0.268665319 | 0.01219528 |
| Direct, low | JL721_12604 | nitrile biosynthetic process | -1.875575764 | 0.0453631 |
| Direct, low | JL721_10006 | NADH dehydrogenase (quinone) activity | 0.667707437 | 0.01550924 |
| Indirect, low | JL721_12728 | COG2931 RTX toxins and related Ca2 -binding proteins | 0.507240974 | 0.14708047 |
| Indirect, low | JL721_4367 | cellular response to paclitaxel | 0.288654707 | 0.01092622 |

Supplemental Table 7. Putative toxin genes.
